# Social marginalization risk and its negative association with socialising preferences in Japanese gamers

**DOI:** 10.1371/journal.pone.0353122

**Published:** 2026-07-09

**Authors:** Brian Confessor, Monica Perusquia-Hernandez, Kongmeng Liew, Hideaki Uchiyama, Naoya Isoyama, Kiyoshi Kiyokawa

**Affiliations:** 1 Division of Information Science, Nara Institute of Science and Technology, Ikoma, Japan; 2 School of Psychology, Speech and Hearing, University of Canterbury, Christchurch, New Zealand; Yale University, UNITED STATES OF AMERICA

## Abstract

For people with a high risk of social withdrawal, such as Japanese “hikikomori” or NEET, socialising with others can be demanding. For some, however, an alternative to direct real-world socialisation comes in the form of videogames. Past research suggests that videogames could help by providing opportunities for virtual social interaction and relational wellbeing management. But would those with a high risk of social marginalization also withdraw from social interaction in games? Past research entertains both possibilities: individuals may avoid socialising in-game as they do offline, but the anonymity of online gaming could afford safer opportunities for social interaction. To better understand the potential of game-based interventions for audiences at high risk of social withdrawal, we investigated gaming preferences of Japanese players (N = 587) with varying levels of NEET-Hikikomori risk. We hypothesized that higher withdrawal risk, measured by the NHR Scale, could either positively or negatively relate to player socialisation and cooperation, as measured by the HEXAD and Game Traits scales. After accounting for age, gender and extraversion, results showed that higher NHR score was associated with lower HEXAD Socialiser, lower HEXAD Philanthropist and lower Game Traits Social orientation scores, suggesting a negative association between NEET-Hikikomori risk and player traits related to socialisation. Furthermore, exploratory analysis showed that a higher NHR score was associated with lower scores in HEXAD Achiever and Game Traits Challenges orientation, suggesting NEET-Hikikomori risk has a negative correlation with challenges and achievement-related gameplay. These findings demonstrate how understanding specific gamer personality types is essential for designing experiences that can act as bridges towards more socialisation, while avoiding the risk of worsening players’ conditions through misaligned design.

## 1. Introduction

Severe forms of social withdrawal, such as hikikomori, have been affecting millions of people in Japan [1]. The term, which describes both the condition and the people afflicted, was popularized by Tamaki Saito as “people who withdraw from social interactions and activities for usually more than six months” (Saito, 2013). Teo and Gaw(2010) [1] define it as a possibly culture-bound syndrome that may meet acceptance criteria as a new psychiatric disorder, characterised by prolonged social withdrawal, and strong avoidance of social situations and relationships. This avoidance causes significant interference in one’s normal, occupational, and social routines, leading to significant social impairment or distress associated with said isolation [2].

While these definitions provide a binary result of ‘hikikomori’ or ‘non-hikikomori,’ the condition can be considered along a continuous spectrum of social marginalization. This is a common approach for understanding psychological disorders (e.g., autism), and is particularly useful for examining individuals’ risks of experiencing that disorder [3]. Accordingly, the NEET-Hikikomori Risk Factors (NHR) Scale [3] notes commonalities in psychological tendencies between NEETs (individuals ‘Not in Employment, Education or Training’ [4]) and hikikomori. It considers both NEETs and hikikomori on a spectrum of psychological tendencies, which are associated with the risk of becoming culturally marginalized. This measurement is based on risk factors related to their lifestyle preferences, thoughts on self-competence, and lack of future ambitions. Our research aims to investigate the psychological dispositions associated with tendencies toward social marginalization. Therefore, we adopt a spectrum definition of social marginalization, rather than stricter, diagnosis-based classifications of forms of social isolation such as hikikomori, which may under-represent marginalization tendencies and risks errors with statistical inference [5].

It is hard to locate, diagnose, investigate the prevalence, and even estimate the number of people who may suffer from social marginalization, given the very nature of the condition. However, reports of various forms of social marginalization and withdrawal have become more frequent over the years, particularly after the COVID-19 pandemic. Despite the initial belief that hikikomori may be a Japan-specific phenomenon, research has suggested that it may be a global health problem [6,7]. The psychological tendencies underpinning hikikomori behaviour were also reported in countries such as Singapore [8], Italy [9], the United States [10], and Malaysia [11]. Additionally, while most studies showed a higher prevalence of this condition in young men [12–17], some studies have reported the opposite trend, such as a 2023 epidemiological survey in Japan, which reported almost no gender differences [18]. Furthermore, cultural differences in Japan may have contributed to this discrepancy, according to Nonaka et al. [19]. Given these contradictory trends, further research is needed to better understand whether age and gender are deciding factors in the hikikomori condition.

In most reports, hikikomori appears to have negative effects on the affected people, their friends, and families [6,20]. For those affected, past research has also extensively shown that their prolonged social isolation may have adverse effects on physical and mental health, increasing the likelihood of problems such as cardiovascular problems, type 2 diabetes, mental health problems, self-harm tendencies, dementia, and early death [21–26].

Given these negative consequences, researchers and the government have been looking for ways to integrate socially marginalized people, such as hikikomori, back into a more social lifestyle, with videogames being a suggested medium towards this goal. For instance, Location-Based Games such as Pokémon GO exemplify ways that player socialisation may be improved by playing certain games. Some researchers have suggested that such games may increase social interaction between players [27], and there have been reports of hikikomori patients who started to go out more often to play the game, and consequently started communicating with other players in the process [28].

Video game preferences might also be linked to personality, and several questionnaires have been developed to tie personality constructs with gaming inclinations in past research [29–31]. Understanding the unique profiles and personality traits of socially marginalized audiences is a prerequisite for designing effective video-game interventions that support resocialisation efforts.

Despite the suggestions of potential game-based approaches for this audience in the literature, there is little empirical evidence of their effectiveness in improving socialisation among socially marginalized audiences. Furthermore, there is little research on what video game elements and mechanics might be appealing to them. To address the current gap in the literature, this study aims to investigate general personality traits and gamer-specific personality types, and to explore the relationships between these two domains. By mapping how broad psychological dispositions correlate with specific gaming inclinations, we aim to provide a theoretical foundation for developing tailored gameplay experiences for socially isolated audiences. Understanding how game-based experiences could encourage safe social interaction is critical, as misaligned interventions risk undermining player wellbeing and potentially worsening their isolation [32].

To better understand the relationship between gamer preferences and psychological traits described by existing tools in the literature, such as the NHR scale [3], the current study focuses on marginalized gamer types within the specific context of Japan. Focusing on a Japanese sample is optimal, as the scales used in this study were specifically developed to capture psychological orientations and triggers specific to Japanese society. Japanese society has pressures on self-competence and future ambitions within a collectivist framework. Furthermore, as extreme social withdrawal was first identified as a “culture-bound syndrome” in Japan [1], this setting provides the most robust environment for understanding how social isolation tendencies may translate into digital behavior. Consequently, this research identifies the specific motivational orientations of socially isolated Japanese players that can be leveraged to develop safer and more effective game-based experiences that encourage socialisation.

## 2. Related work

### 2.1. Video games and social isolation

Video games have demonstrated significant potential to improve various aspects of life and foster social connections among diverse audiences. For instance, a 1981 study found that exposing students to games with cooperative elements increased prosocial behavior and sharing [33]. However, the potential efficacy of games as therapeutic resocialisation tools is fundamentally tied to how well the game’s environment aligns with players’ specific psychological dispositions and personality traits.

In specific demographic contexts, exergames have also been used to induce beneficial lifestyle choices, such as increasing exercise frequency and social interaction among elderly players [34]. Most notably, location-based games like Pokémon GO can satisfy various player needs, including physical activity, escapism, and social interaction [35]. Using such games in the post-COVID era has helped players build new relationships, which in turn has led to increased engagement with the medium [36]. There are also documented reports of extremely socially withdrawn individuals who began leaving their homes and communicating with others specifically to play these games [28].

Furthermore, for those who feel a deep need to belong despite their isolation, genres such as Visual Novels can potentially satisfy the desire for social connection through “narrative collective-assimilation” [37]. The appeal of this genre could suggest that specific player traits — such as an orientation towards narrative elements and low-risk social cues — could be leveraged by developers to create ‘entryway’ experiences that satisfy players’ need for belonging, without the immediate pressure of direct social confrontation.

Despite these benefits, the relationship between social isolation and gaming is often overshadowed by clinical risks and negative stereotypes. Individuals at high risk of social marginalization often report high frequencies of gameplay, particularly in Role-playing, Action, and Simulation genres [38]. Past research has linked these high-withdrawal symptoms to increased risks of Internet Gaming Disorder (IGD), which is a condition listed on the Diagnostic and Statistical Manual for Mental Disorders (DSM-5) as a “persistent and recurrent use of the internet to engage in games (8-10h of gameplay per day, and at least 30h per week), often with other players, leading to clinically significant impairment or distress.”. This association is more pronounced with longer gameplay times [39]. Impulsivity problems were also observed in some cases. Impulse control disorders were one of the comorbidities found in a Japanese hikikomori prevalence study [12]. Increased reports of gaming addiction and internet usage have also been specifically noted in Japanese populations experiencing social withdrawal [40]. Additionally, social motivations themselves can serve as predictors for online game addiction [41]. The very communities meant to provide connection can also be detrimental; players who perceive their gaming communities as toxic report higher loneliness and lower “relatedness” satisfaction, creating a vicious cycle that further draws vulnerable players away from healthy interaction [42].

However, the deep engagement that socially marginalized audiences already have with this medium provides a crucial opportunity for intervention. Because these individuals often consume media for long periods and are already familiar with various game genres [38], video games can serve as the most effective “gate” to facilitate social interaction. For instance, location-based games are seen by researchers as a potentially useful tool for hikikomori resocialisation because they could help “rescue” shut-ins from their isolated world, by improving socialisation through playing [27]. Even when physically withdrawn, these individuals may still possess “digital social capital” — the perceived social resources obtained through online interactions—and remain willing to engage socially in digital spaces [43]. By understanding the specific personality traits of socially isolated audiences [30], game developers can design tailored, safe virtual environments for these audiences that can leverage the existing passion for games to foster meaningful, healthy relationships and potentially reduce isolation risk.

### 2.2. Social isolation, personality traits and player motivations in games and online media

Different people have different preferences and tastes, and the same is true for preferred game genres and personality-based inclinations. Past research has extensively investigated possible connections between a player’s preferences for video games and constructs related to personality traits. One construct often used was the Big 5 Personality Traits Model [44,45], one of the most widely used frameworks for understanding personality traits. Several scales that correlate player-preference constructs with the Big 5 were also developed [46–48]. By analyzing the personality traits and motivations of a given audience, it becomes easier to understand which game elements might appeal to them. To understand the personalities of gamers when it comes to their player preferences, we can utilize measurements such as the Game Traits Scale (GTS) [31], which quantifies players’ preferences towards player-specific dispositions on five traits: Aesthetic, Narrative, Goal (directedness), Social (orientation), and Challenge.

Similarly, the 24-item HEXAD Scale [30], adapted from the HEXAD Framework [29], measures user motivations in gaming, focusing on gamified applications. It maps game motivations across six dimensions: Achiever, Philanthropist, Disruptor, Socialiser, Player, and Free Spirit. Gamified applications, unlike traditional games, employ gamification [49]. Gamification is the inclusion of game-like elements in non-game applications and systems, used to increase user motivation and enable changes in attitude and behaviour [50].

Research has shown possible correlations between personality traits and social isolation. For instance, Bonnaire and Roignot [51] investigated the relationships between personality dimensions, coping strategies, and the hikikomori condition, and found that introversion (or low extraversion) was positively correlated with the hikikomori levels. Additionally, Lounsbury and colleagues [52] found that low conscientiousness was correlated with Futoko (school absenteeism); this behavior could be understood as a possible precursor of social withdrawal for adolescents [53].

Similarly, Judge and colleagues [54] found that low conscientiousness was correlated with job absenteeism. Liew and colleagues [8] investigated the social marginalization risks in Singapore using the NHR Scale [3], and found that extraversion was negatively associated with the NHR’s Lack of Self-Confidence factor, while the Unclear Ambitions factor had a negative association with conscientiousness.

Furthermore, compulsive internet usage, or internet addiction, has been extensively shown to be related to introversion (or low extraversion) and socially isolated behaviour [39,40,55–58]. This compulsive use may partly be due to the ease of anonymity online. Past research [59] investigated the factors that lead people to seek anonymity online. For some, prior negative experiences were one of the reasons they sought anonymity, and, by being anonymous, participants reported it was easier to avoid disliked people and to build new relationships. Some also describe feeling more relaxed, comfortable, and free to express their views in an anonymous environment. Although they argue that discouragement in anonymity can reduce malicious behaviour, they also acknowledge a paradox. The same anonymity can foster helpful, creative, and harmless online activities that people may be interested in pursuing, such as forming new relationships with less fear of judgment. Similarly, a 2021 study [60] proposed that online social interactions may help socially isolated people maintain social connections despite physical isolation.

## 3. Research questions and hypotheses

Our goal was to investigate whether there was a relationship between players’ social marginalization risk levels and gamer personality traits. We posit that these relationships could also be explained through introversion (or low extraversion), which has been linked to both player motivations [29,30,46–48], and social isolation tendencies [8,51,56].

To quantify gamer traits, our study used the HEXAD Scale [30] and the GTS [31] to measure player motivations, as they were already validated in the literature and linked to personality traits. Therefore, we focused on constructs within each scale related to introversion/extraversion, as low extraversion scores have been associated with a higher risk of social marginalization [8].

High extraversion was associated with the Philanthropist and Socialiser Gamer Types in HEXAD [29,30], and the Social Orientation aspect of the GTS [31]. The HEXAD Philanthropist construct is interested in being helpful and altruistic towards others during gameplay and is also associated with extraversion. The HEXAD Socialiser and GTS Social Orientation constructs are related to an interest in socialising with other players in video games and gamified applications. Given that socially isolated people typically exhibit high introversion (low extraversion), low self-esteem, and overall low wellbeing, we hypothesize that those at high risk of social marginalization would be less interested in helping other players in-game (H1). Therefore, their Philanthropist score would be lower.

When analysing accounts in the literature, it becomes hard to gauge the interest of socially marginalized people in socialising with others and how that might affect their overall desire to socialise. Given the introverted nature of social isolation, it seems logical at first glance that those at high risk of social marginalization might be more averse and less interested in fostering social relationships, both online and offline. Those with a substantial risk of social marginalization might then score lower on gamer preference constructs related to socialisation, which are related to high introversion (or low extraversion). Therefore, we hypothesized that those with higher marginalization risk could score lower in the HEXAD’s Socialiser User Type (H2a), as well as in the GTS’s Social Orientation (H3a).

Despite this, we see evidence of interest in socialisation in some form among socially isolated people, as shown in studies of hikikomori players [27,39]. As social marginalization is tied to low self-esteem in their offline social interactions [3], the anonymity and the less judgmental environments that video games provide could serve as a protective layer for socially marginalized players to foster social connections more easily [59,60]. Additionally, the established correlation between hikikomori and IGD [39], as well as the relationship between social motivations, impulsivity, and game addiction [41], could suggest that those who are socially marginalized are more likely and willing to forge social connections through some media such as Mass-Multiplayer Online (MMO) games. This suggests a possible positive correlation between a person’s tendencies toward isolation and constructs related to socialisation. In other words, there could be a positive relationship between social marginalization risk and the HEXAD Socialiser User Score (H2b), and between social marginalization risk and the GTS Social Orientation (H3b).

Given this dichotomy in the literature, it seems plausible that higher social marginalization risk could be either positively or negatively correlated with socialisation constructs, and therefore, we investigated both competing hypotheses. In summary, we hypothesize that:

**H1**: Social marginalization risk is negatively correlated with the HEXAD Scale’s Philanthropist User Type.

**H2a**: Social marginalization risk is negatively correlated with the HEXAD Scale’s Socialiser User Type.

**H2b**: Social marginalization risk is positively correlated with the HEXAD Scale’s Socialiser User Type.

**H3a**: Social marginalization Risk is negatively correlated with the GTS’s Social Orientation.

**H3b**: Social marginalization Risk is positively correlated with the GTS’s Social Orientation.

## 4. Methods

### 4.1. Ethics statement

The project hypotheses and analysis methodology were pre-registered on the Open Science Framework prior to data collection. This study received approval from the Institutional Review Board of our local institution (IRB approval number 2022-I-25). Written informed consent was obtained from all participants prior to the participation of the study.

All materials, data, and analyses were conducted with R for data formatting and analysis (version 2023.03.1 + 446 was downloaded from https://www.r-project.org/); we utilized the ‘readxl’ package for library import, ‘tidyverse’ for plotting, ‘moments’ to calculate skewness and kurtosis, ‘lm.beta’ to calculate standardized β coefficients, ‘effectsize’ to interpret the *R*^2^ results, and ‘xtable’ and ‘knitr’ for table building. The source code and analysis are publicly available on our OSF repository, (available through https://osf.io/wp2rh/overview?view_only=0c2e804fc4d341b0b18801226c1ac48b).

### 4.2. Participants

An a priori statistical power analysis was conducted using the negative correlation between extraversion and NHR [8] to determine the sample size in G*Power. Five predictors, one for each hypothesis, were used with a Squared Multiple Correlation (*p*2) of 0.253, an Effect size (*f*2) of 0.034, and an α value of 0.05. This suggested a minimum sample of N = 65. However, the prevalence of people with a high risk of social marginalization in the general population can be relatively low, as was seen in examples such as past research on hikikomori prevalence [12]. Furthermore, Uchida and Norasakkunkit [3] consider 104 points to be the cut-off limit between “High NEET-Hikikomori Risk” (High NHR) and “Low NEET-Hikikomori Risk” (Low NHR). Considering this cut-off point and the difficulty in contacting socially marginalized people, as well as our goal to better understand gamer traits of players with a high risk of social marginalization, we aimed to get as many responses as possible.

Six hundred and seventy-six participants were recruited using the Crowdworks online platform (https://crowdworks.jp/) that redirected them to an online survey, which collected data in two rounds, over a span of two weeks in February 2023. The first round, which lasted for a day, contained the original Japanese text and a translation into English language. However, considering that most eligible participants would not need this translation, it was decided that the translation was not contextually relevant, and thus removed it in the second round, which was available for new participants for the following 13 days.

Participants were given detailed written instructions about the questionnaire presented and provided informed written consent by choosing to participate in the survey. All participants were 18 or older, and each received 200 JPY for completing the survey. The survey was open to people with Japanese nationality and residence who were 18 or older and had previous experience with video games.

**Fig 1:**
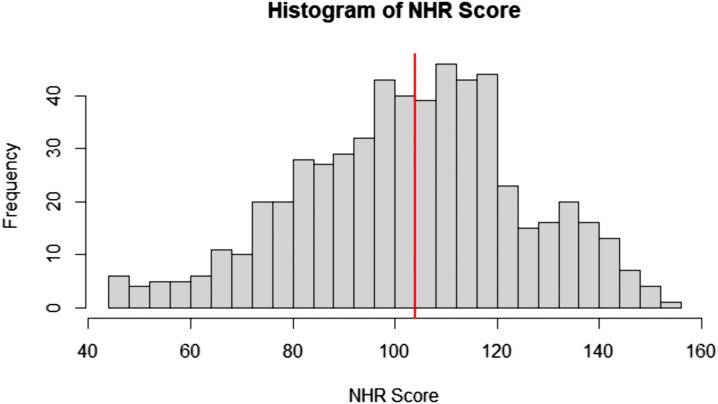
Distribution of the NHR score among the participants, after exclusion criteria were applied. The X-axis represents the range of the NHR score, while the Y-axis represents the number of participants that were in a given range. The red vertical line in the graph represents the 104-point cutoff between high and low NEET-Hikimomori Risk [3].

Exclusion criteria included having answered the questionnaire more than once (in the same round or across both survey rounds), failing at least one of two attention checks, leaving before fully completing the questionnaire, and not being born and raised in Japan. Of our original 676 participants, 89 had invalid responses according to our exclusion criteria, and were thus excluded from the analysis. This resulted in a final N = 587 participants ($M_{a}ge$ = 39.3, SD = 9.13; 50.26% men, 48.72% women, 1.02% chose not to answer). The distribution of NHR scores (M = 103.4, SD = 22.03) from these 587 participants can be seen in Figure 1. After exclusions, 52.3% of participants were considered “High NHR”, according to the cut-off point proposed in previous literature [3]. The average completion time of the questionnaire was around 32 minutes.

To address the potential bias of convenience sampling and ensure the quality of our data, we implemented the validated NEET-Hikikomori Risk (NHR) Scale to measure the hikikomori risk of participants. We found that results ranged between 44 and 153 points, from mild to more extreme risk of social isolation. While recruitment was conducted via convenience sampling, the use of the validated NHR Scale allowed us to measure the risk levels within this specific digital demographic. Our sample mean (M = 103.4) sits just below the proposed 104-point cutoff for ‘High Risk’ [3], resulting in a distribution where 52.3% of participants are classified as high-risk. This distribution is consistent with our recruitment strategy, which specifically targeted individuals with gaming experience on a crowdsourcing platform —-two factors known to correlate with the NEET-hikikomori lifestyle. [39]. Consequently, our sample aligns with contemporary research showing that the hikikomori phenomenon increasingly affects a diverse, middle-aged digital workforce across genders. [61]

### 4.3. Measurements and procedure

We used the GTS and HEXAD to measure inclinations toward gaming styles and preferences. The constructs present in both scales are associated with personality traits described by the Big 5 Personality Traits Model [44,45]. The gamer traits described by the GTS are described in Table 1, while the definitions of the HEXAD dimensions can be seen in Table 2. The constructs in the two scales are not orthogonal, and some dimensions overlap conceptually, such as the GTS Social Orientation and the HEXAD Socialiser trait.

**Table 1:** Definition of Player Orientations as described by the Game Traits Scale.

Player Orientation	Description
Aesthetic	Enjoyment of aesthetic game experiences, such as world exploration and appreciation of the quality of sounds, graphics, or art. Players with higher Aesthetic scores tend to be more open to experience.
Narrative	Enjoyment towards complex narratives and stories within games. Players with higher Narrative scores are usually more open to experience and introverted.
Goal	Enjoyment in completing a game’s goals and collectibles. Players with higher Goal scores tend to be more neurotic and conscientious.
Social	Enjoyment towards social features and interactions. Higher scores are associated with players who are more agreeable, extroverted, and less neurotic.
Challenge	Enjoyment towards more challenging and difficult games. More neurotic players usually have lower Challenge orientation scores.

**Table 2:** Definition of User Types as described by the HEXAD Scale.

HEXAD Type	Description
Achiever	Positively correlated with Conscientiousness. Motivated by competence, users who wish to progress in systems by completing challenges and tasks.
Philanthropist	Positively correlated with Extraversion, Agreeableness, Openness, and Conscientiousness. Motivated by purpose. Altruistic users who are willing to help without expecting compensation.
Disruptor	Negatively correlated with Neuroticism. Motivated by triggering change, these users like to test the system’s boundaries. They are motivated by trying to disrupt the system, either positively or negatively.
Socialiser	Positively correlated with Extraversion and Agreeableness. Motivated by relatedness, these users create social connections and interact with others.
Player	Positively correlated with Conscientiousness. Motivated by extrinsic rewards. Users are driven by the opportunity to earn rewards in systems.
Free Spirit	Positively correlated with Openness and Extraversion, negatively correlated with Neuroticism. Motivated by autonomy. Users are interested in freely exploring and expressing themselves within the system.

Participants’ personality traits and psychological tendencies were also assessed to better understand their potential social marginalization. Rather than behavioural measures and binary diagnoses employed to identify hikikomori people, we focused on psychological risk tendencies, following common practices from past research. For that, we used the NEET-Hikikomori Risk (NHR) scale [3], a 27-item survey that has been externally validated in showing strong associations with other cognitive and behavioural indicators of social isolation [62]. This scale was theoretically validated in accordance with the Japanese government’s classification of NEET and Hikikomori. It was also shown to sufficiently discriminate between these groups and lesser degrees of marginalization in Japanese society. Furthermore, scores in the NHR Scale were strongly and inversely associated with general wellbeing, job satisfaction, close relationships within Japanese society, and self-reported health status. We therefore found that it could accurately estimate Japanese participants’ risk of social marginalization in Japan.

The survey was presented to participants through the SurveyMonkey (https://www.surveymonkey.com/) platform. The survey included the Ten-Item Personality Inventory (TIPI-J) scale [63], the NHR Scale, the HEXAD and the GTS, and other sociodemographic questions. In many personality inventories, including the TIPI-J, extraversion and introversion are measured on the same dimension, with high scores indicating high extraversion and low introversion, and low scores indicating low extraversion and high introversion.

We used validated Japanese versions of the TIPI and NHR scales and translated the HEXAD and GTS. Two native Japanese-speaking bilinguals independently translated the scales into Japanese. Then, the Japanese translation was back-translated to English by two different bilingual translators. Discrepancies were resolved through discussion.

Our survey examined tendencies toward social marginalization among Japanese people; therefore, we focused on individuals raised in and currently residing in Japan. Attention checks were included in the questionnaire (for example, “Please mark ‘Completely Agree’ for this question”), and participant answers were considered valid only if at least 50% of the attention checks were answered correctly. Finally, since this study focused on personality traits in video games, our recruitment post on Crowdworks specifically asked for participants with gaming experience.

### 4.4. Analysis method

As HEXAD and GTS have not been previously used in Japan, a Confirmatory Factor Analysis (CFA) was conducted to determine their construct validity. Next, we analysed the relationship between the NHR score, which was the outcome (dependent) variable for all of our models, and each of the three predictor (independent) variables: HEXAD Philanthropist Score; HEXAD Socialiser Score; and GTS Social Orientation Score. These three variables are related to our hypotheses, and were thus dubbed “Hypothesis Variables”. For each predictor, we conducted three steps in the analyses: (a) Baseline Analysis: A linear regression analysis between NHR Score and one predictor variable; (b) Age and gender as covariates: Controls (covariates) for participants’ age and gender were included alongside the Baseline model. Given that some types of social marginalization, like hikikomori, may be more prevalent among male youth and young adults [12,14–17], accounting for such variables could be relevant when interpreting our results; (c) Extraversion: As Extraversion could be a confounding factor, Extraversion was also included on the benchmark model as a covariate. As the HEXAD Socialiser, GTS Social Orientation, and NHR scores had negative correlations with extraversion [3,29–31], there was a possibility that extraversion could be a confounding factor in any identified relationships between the variables. To examine the robustness of any observed effects of HEXAD Socialiser and GTS Social Orientation scores on NHR, beyond the effect of extraversion, these constructs were also included as covariates, alongside age and gender.

We also conducted exploratory analyses between NHR and the remaining factors of the HEXAD (Achiever, Disruptor, Player, and Free Spirit) and GTS (Aesthetic, Narrative, Goal, and Challenge). These remaining variables were dubbed “Exploratory Variables”. Despite not being directly related to our main hypotheses, understanding the relationships between social marginalization risk and the Exploratory Variables would allow follow-up research to examine potential outcomes in optimising game-based interventions for socially marginalized audiences.

## 5. Results

The interpretation of the effect size results in our analyses followed Cohen’s understanding of effect size magnitudes [64].

### 5.1. Construct validity

Multiple fit indices indicated a poor fit, $\chi^{2}$(237) = 1287, *p* < .001, Comparative Fit Index (CFI) = 0.834, Tucker-Lewis Index (TLI) = 0.806, Root Mean Square Error of Approximation (RMSEA) = 0.087. However, for a model that included only the Philanthropist and Socialiser factors, fit indices generally indicated an acceptable fit, $\chi^{2}$(19) = 85.7, *p* < .001, CFI = 0.976, TLI = 0.965, RMSEA = 0.077. Reliability was also high for the HEXAD Philanthropist (Cronbach α = 0.84) and Socialiser (Cronbach α = 0.90) factors.

Multiple fit indices indicated a poor fit for the full five-factor Game Traits Scale, $\chi^{2}$(265) = 1445, *p* < .001, CFI = 0.881, TLI = 0.866, RMSEA = 0.087. However, for a model with only the Social Orientation (related to our hypotheses), fit indices generally indicated an acceptable fit, $\chi^{2}$(5) = 63.8, *p* < .001, CFI = 0.977, TLI = 0.954, RMSEA = 0.142. Reliability was also high for the Social Orientation factor (Cronbach α = 0.92).

The results suggest that, while the HEXAD Scale and GTS may have poor construct validity in the Japanese context, the factors of interest for our hypotheses (HEXAD Philanthropist and Socialiser; Game Traits Social Orientation Score) show acceptable construct validity and strong reliability in our Japanese sample.

For external validity, we correlated these scales with extraversion. Consistent with past research, NHR was negatively correlated with extraversion (r=−0.52, *p* < .001), but HEXAD Philanthropist (r = 0.35, *p* < .001), HEXAD Socialiser (r = 0.49, *p* < .001), and Gamer Traits Social Orientation Scales (r = 0.25, *p* < .001) were positively correlated with extraversion. These suggest that previous research linking HEXAD Socialiser, HEXAD Philanthropist, and GTS Social scores with extraversion in Western samples was replicated in our Japanese sample, as evidence of external validity for these constructs.

### 5.2. Hypothesised analyses

In Model 1, we first executed three separate regression models to investigate possible associations between the NHR Score (dependent variable; M = 103.4, SD = 22.03) and the Hypothesis Variables (Table 3). A significant negative relationship was observed between NHR and the HEXAD Philanthropist Score, **supporting H1**; as well as NHR and HEXAD Socialiser Scores and Game Traits Social Orientation scores, **supporting H2a and H3a**. In other words, **higher NHR score was associated with lower HEXAD Socialiser, lower HEXAD Philanthropist, and lower GTS Social Orientation scores.**

**Table 3:** Model 1 – Hypothesis Variables – Benchmark Analysis.

Linear Model: NHR vs. Hypothesis Variables 1					
Indep. Variable	Standardized β	95%CI-L	95%CI-U	Adj. *R*^2^	*p*-value
H. Philanthropist	−0.416	−0.797	−0.035	.172	*p* < .001
H. Socialiser	−0.532	−0.835	−0.228	.281	*p* < .001
G. T. Social	−0.208	−0.456	0.040	.042	*p* < .001

*Note.* α=0.05.

These effects were significant even after the inclusion of age and gender (Model 2, Table 4) and Extraversion (Model 3, Table 5) as covariates. We noticed a slight decrease in Standardized β coefficients for models that included extraversion. Still, our variables of interest, HEXAD Socialiser, HEXAD Philanthropist, and GTS Social Orientation Scores, were significantly associated with NHR, above and beyond the effect of extraversion.

**Table 4:** Model 2 – Hypothesis Variables – Covariate Analysis (Age+Gender).

Linear Model: NHR vs. Hypothesis Variables 2					
Indep. Variable	Standardized β	95%CI-L	95%CI-U	Adj. *R*^2^	*p*-value
H. Philanthropist	−0.426	−0.800	−0.053	.218	*p* < .001
H. Socialiser	−0.546	−0.840	−0.253	.335	*p* < .001
G. T. Social	−0.265	−0.512	−0.017	.104	*p* < .001

*Note.* α=0.05.

**Table 5:** Model 3 – Hypothesis Variables – Covariate Analysis (age+gender+Extraversion).

Linear Model: NHR vs. Hypothesis Variables 3					
Indep. Variable	Standardized β	95%CI-L	95%CI-U	Adj. *R*^2^	*p*-value
H. Philanthropist	−0.282	−0.639	0.075	.370	*p* < .001
H. Socialiser	−0.388	−0.703	−0.072	.415	*p* < .001
G. T. Social	−0.121	−0.348	0.105	.313	.001

*Note.* α=0.05.

### 5.3. Exploratory analysis

Analogous to the Hypothesis Analysis section, the relationship between the NHR Score and each of the remaining variables of the HEXAD and the GTS was explored in separate linear regression models (Model 4, Table 6). We also conducted subsequent analyses that included age and gender as covariates (Model 5, Table 7). Unlike the hypothesis variables, extraversion has only been previously associated with the HEXAD Free Spirit User Type and the GTS Narrative Orientation, and thus has no relation to most of our exploratory variables. We reasoned that extraversion would have a lesser confounding effect on NHR; therefore, we excluded it from the Exploratory Analyses. The following analyses follow the same rules as the Hypothesis Analyses regarding variable normalization.

**Table 6:** Model 4 – Exploratory Co-variate Analysis 1.

Linear Model: NHR vs. Exploratory Variables					
Indep. Variable	Standardized β	95%CI-L	95%CI-U	Adj. *R*^2^	*p*-value
H. Free Spirit	−0.340	−0.777	0.098	.114	*p* < .001
H. Achiever	−0.441	−0.844	−0.039	.193	*p* < .001
H. Disruptor	0.104	−0.328	0.536	.011	.009
H. Player	−0.223	−0.689	0.243	.048	*p* < .001
G. T. Aesthetic	0.033	−0.275	0.341	−.001	.426
G. T. Narrative	−0.060	−0.341	0.222	.002	.149
G. T. Goal	−0.031	−0.339	0.276	−.001	.452
G. T. Challenge	−0.184	−0.480	0.112	.032	*p* < .001

*Note.* α=0.05.

**Table 7:** Model 5 – Exploratory Co-variate Analysis 2.

NHR vs. Exploratory Variables w/ age and gender as covariates					
Indep. Variable	Standardized β	95%CI-L	95%CI-U	Adj. *R*^2^	*p*-value
H. Free Spirit	−0.357	−0.784	0.071	0.165	*p* < .001
H. Achiever	−0.438	−0.832	−0.044	0.230	*p* < .001
H. Disruptor	0.085	−0.346	0.516	0.045	.039
H. Player	−0.241	−0.700	0.219	0.096	*p* < .001
G. T. Aesthetic	−0.029	−0.345	0.287	0.039	.499
G. T. Narrative	−0.104	−0.385	0.176	0.048	.012
G. T. Goal	−0.052	−0.326	0.251	0.041	.205
G. T. Challenge	−0.215	−0.510	0.079	0.083	*p* < .001

*Note.* α=0.05.

Among the variables in Model 4, the HEXAD Free Spirit, HEXAD Player, and GTS Challenge factors showed significant negative relationships with the NHR Score, marked by weak effect sizes. The HEXAD Achiever variable, similarly, showed a significant negative relationship with the NHR Score, with a moderate effect size. The HEXAD Disruptor showed a significant positive relationship to the NHR score, but with a weak effect size. Aside from the above, the remaining constructs (GTS Aesthetic, Narrative, and Goal orientations) did not show significant results. These results were robust in Model 5 after including age and gender as covariates. Moreover, Model 5 showed a significant but weak negative relationship between NHR Score and the GTS Narrative orientation.

In other words, **higher NHR score was associated with lower scores in the HEXAD Free Spirit, HEXAD Achiever, HEXAD Player, Game Traits Narrative and Game Traits Challenge constructs, even after accounting for age and gender.**

## 6. Discussion

We investigated the relationship between a person’s social marginalization risk (NHR) and their gamer traits. Establishing these relationships provides the empirical clarity necessary to design safe, trait-aligned gaming experiences that can effectively serve as an engaging ‘entryway’ towards improving players’ socialisation.

Our results showed that a higher NHR score was associated with lower social inclinations in video games (as reflected in lower HEXAD Socialiser and Game Traits Social scores) and lower inclinations to help others (HEXAD Philanthropist). These results were robust even after including age, gender, and extraversion as covariates, demonstrating that the negative associations remained statistically significant after accounting for demographics and introverted personality dispositions in our Japanese sample.

The moderate negative association between the NHR Score and the HEXAD Philanthropist Score supports H1. This finding is consistent with results from previous research conducted in Hong Kong [65], which showed that social support was positively associated with the Philanthropist construct during the validation of the Chinese 12-item version of the HEXAD Scale. This suggests that philanthropic orientations in games may be less interesting for people with higher risk of social marginalization. However, further confirmatory testing would be needed to determine whether these psychological associations remain stable across different regional contexts.. One possible reason is that the psychological consequences of social marginalization — such as social exclusion or loneliness — have been shown to reduce prosocial motivation and helping behavior in past research. For example, experimentally induced social exclusion reduced helping and cooperation in behavioral tasks [66], while exposure to loneliness cues decreased contributions in public goods games [67]. These findings suggest that socially isolated people may be less inclined to engage in helping actions, even when social rewards are available.

The moderate negative association between the NHR Score and the HEXAD Socialiser Score supports H2a and rejects H2b. Similarly, the weak negative association between the NHR Score and the Game Traits Social orientation supports H3a and rejects H3b. Both constructs in these hypotheses deal with socialising with other players in games. These results are also corroborated by previous results [65], which showed a positive relationship between social support and the HEXAD Socialiser construct. One potential implication of these results is that emerging social withdrawal tendencies are not limited to in-person social interactions, but appear to generalise to in-game interactions as well. This contrasts with psychopathological conditions such as social anxiety, where avoidance of social interactions is associated with sensitivity to negative in-person emotions and negative facial expressions [68]. In these conditions, the anonymity of online multiplayer interactions in games has been shown to encourage online social interactions in players [69]. Despite several overlaps between social anxiety and social withdrawal as comorbid conditions [70], our results show differing trajectories of online tendencies: high-risk players still appear to shy away from social interaction in online games. This adds to a growing body of literature identifying how social withdrawal may exhibit distinctive behavioural trajectories compared with social anxiety (e.g., [71]).

These results, therefore, suggest that socially-oriented gameplay preferences by themselves may not be particularly interesting to players with a tendency towards social marginalization, or that these players choose not to engage much with social elements.

The moderate negative association between the NHR Score and the HEXAD Philanthropist, HEXAD Socialiser and Game Traits Social scores all suggest an inverse relationship between preferences towards social-based gameplay and social marginalization risk. This is consistent with the definitions for the HEXAD Philanthropist and Socialiser constructs [29,30] and the Game Traits Social orientation [31], all of which are negatively associated with Extraversion. Furthermore, the HEXAD Scale validation made by Fong et al. further corroborated that social support was positively associated with the Philanthropist construct [65]. This comparison is grounded in the fact that high NHR scores are fundamentally characterized by a deficiency in social resources and an inverse relationship with close social ties. Thus, the negative association identified in our study aligns with the positive association found in prior work: while social support encourages prosocial traits, the risk of marginalization appears to inhibit them.

It is important to note, however, that this observed preference for lower social engagement should not be interpreted solely as a psychological deficit. Instead, such tendencies may represent a proactive coping strategy used by high-risk players to manage their emotional well-being in digital environments, particularly those that can lead to stressful situations. Previous research [42] found that “players who perceived their gaming communities as toxic also reported higher loneliness and lower need satisfaction of relatedness”. Considering these results, gravitating toward more solitary forms of engagement could be the way some players avoid the ‘vicious cycle’ of toxicity found in some online gaming communities. One possible medium that could offer ‘low-risk’ experiences are narrative-driven Visual Novels, which remove the immediate pressure of direct social confrontation and the stress of negative feedback, while still offering the possibility of forming parasocial relationships with the characters in the stories. This allows players to feel like they are maintaining their ‘digital social capital’ [43] while protecting themselves from further social impairment.

On the other hand, past research has shown that digital and online approaches to reach this audience are promising. A 2015 survey [13] found no significant difference in digital social capital between control participants and people with severe social withdrawal. Digital social capital refers to the perceived social resources (e.g., support, belonging, reciprocity, informational access) that individuals obtain through online social networks and digital interactions [43]. This suggests that socially isolated people can and may still be willing to engage in social interactions digitally, despite being physically or socially withdrawn offline. Another study [72] utilized social media to successfully reach and engage socially withdrawn youth in China. Through online recruitment and survey participation, the study found that this population remains active in digital spaces and responsive to online communication channels, even if they avoid face-to-face interaction. Considering this possible interest in digitally mediated interaction, video games may serve as a medium to bridge online relationships for this group and potentially reduce the risk of isolation. However, careful attention must be paid to the design of these games to capture the attention of players with a high risk of social marginalization.

The potential benefits of social elements in video games have also been well established. When discussing high-risk players, past reports suggested that even those with severe social isolation, such as hikikomori, could be interested in using games such as Pokémon GO for social connection [28]. Additionally, past research suggests that games such as Pokémon GO can be used to fulfill social needs and generate positive feelings through social interactions [35,36,73]. One research [73] in particular noted that social interactions caused by playing could bring affective benefits to players, especially for those with noxious moods, such as those with depressive symptoms. Therefore, it is necessary to weigh the risk of fueling a possible gaming addiction with the possible benefits provided by social interactions fostered by an intervention game with social features. By designing the gaming experience to avoid common triggers that may be associated with gaming addiction, such as controlling play time and limiting in-game purchase options [74], game developers can steer away from addiction risks and focus the gameplay experience on the beneficial aspects of socialisation games. Furthermore, if social elements are included in game-based interventions and adequate filters and control systems are put in place to address player toxicity, high-risk players may also reap the positive feelings that can arise from social interactions.

The Exploratory Analysis also found negative associations between NHR level and constructs related to game challenges (HEXAD Achiever and Game Traits Challenge Orientation). Lower scores in these constructs are related to high Neuroticism and low Conscientiousness, which are also related to possible precursors to hikikomori [52,54]. People who score high on these constructs are usually interested in proving themselves by facing complex tasks and challenges. In contrast, those with a higher risk of NEET-hikikomori may be less likely to persist and fully complete objectives and difficult challenges in certain games and gamified applications, which could decrease player engagement. This is corroborated by previous studies showing that individuals with higher hikikomori risk were less likely to persist on challenging tasks when faced with negative feedback [75].

Weak but significant relationships were also found in the HEXAD Free Spirit and Player Scores models, with higher NHR scores being associated with lower Free Spirit and Player scores. The HEXAD Free Spirit User Type is positively correlated with openness and extraversion, and negatively correlated with Neuroticism. It is related to being more open-minded to new experiences, and freedom to creatively express one’s feelings while creating and exploring within systems. These results corroborate previous research [76], which conducted a case study with 35 socially withdrawn young participants, who frequently reported feelings of inhibition of self-expression. Our results suggest that participants with a higher risk of social marginalization, who seem more introverted and possibly more self-conscious, could have a harder time expressing their thoughts and opinions. This also aligns with previous studies reporting negative relationships between school/job absenteeism (possible precursors of social isolation) and Openness [52] and Neuroticism [54].

This lack of desire —or perhaps a feeling of inability— to express individual desires reflects the collectivist landscape of Japan, where the societal norm is to not stand out and follow rules. These societal expectations can also fuel similar behaviour in the game world, with games also becoming a reflection of its cultural environment, discouraging deviance from societal norms [77]. Past research has also shown that marginalized youth are averse to being interdependent and conforming to prevalent Japanese social pressures [78]. One explanation for this could be that the cultural influences of collectivism are stronger and more persistent in the psyche of socially marginalized people than the individual’s motivation towards personal freedoms.

Given the apparent aversion of high-risk players to creative and expressive gameplay, game developers who still wish to develop these features for socially marginalized audiences may benefit from creating a safe, reassuring gaming environment. This could make players feel more at ease and encouraged to express themselves without fear of negative repercussions. This is akin to previous research that utilizes similar ideas of environments to foster self-expression in users [79].

### 6.1. Design suggestions and implications for interventions

To move from psychometric profiles and traits explored in this study to more practical interventions, our findings can be interpreted into more grounded game design suggestions, using the **Self-Determination Theory** (SDT) as a foundation for our suggestions [80]. The SDT is a macro-theory of human motivation which posits that for individuals to experience high-quality engagement and well-being, three basic psychological needs must be satisfied: Autonomy (the feeling of agency and choice), Competence (the experience of mastery and effectiveness), and Relatedness (the sense of connection and belonging with others). [80] Thus, we posit that the game design elements we suggest should fulfill players’ three core needs, as described by the SDT. For individuals with high marginalization risk, who seem to show an aversion towards traditional challenges and social situations that may create social pressure, we propose that the game design should focus on:

- **Steady, RPG-like progression and ’simple mechanics’**: Mechanics should be slowly introduced to avoid sharp increases in difficulty. This allows players to experience a sense of growth and mastery without the difficult hurdles and negative feedback that frequently lead to disengagement in this population, and thus can foster their Competence needs.
- **Focus on cooperative gameplay rather than competitive:** This can help with players’ Relatedness needs, by allowing players to interact with one another, while mitigating the fears of being a ’nuisance’ or facing toxic interactions, which currently act as a barrier to social participation for high-risk players. Similar to the RPG-like mechanics, social elements should also aim for a slow-start entry point, being introduced slowly to avoid any sudden negative feedback, and progress towards long-term interaction.
- **Short, flexible play sessions**: This provides players with the agency to manage their own visual or physical fatigue, ensuring the digital environment remains an inviting ‘gate’ rather than a source of stress. This helps to avoid a buildup of stress over long play sessions, while also fostering players’ Autonomy needs.

## 7. Limitations and future studies

Recruiting participants who are at high risk of social marginalization is a challenge due to their reclusive nature. This led to our decision to conduct an online survey where, given their lifestyles, individuals with high NEET-Hikikomori risk and corresponding social withdrawal tendencies may see crowdsourcing websites as a source of income and may be better represented on these platforms. Moreover, our participant recruitment explicitly sought individuals with experience with video games, which has been shown to correlate with hikikomori behaviour [39]. This may help explain our relatively elevated proportion of high-risk participants (52.3% of our participants were considered “High NHR”), compared to 21.7% reported by Norasakkunkit & Uchida [75] from a Japanese University sample, and 22.3% by Lin and colleagues [62] in a Singaporean community sample.

The ‘borderline’ nature of our sample mean (M = 103.4) compared to the 104-point cutoff from the literature [3] provides a unique opportunity to investigate the threshold of social withdrawal risk. Unlike clinical samples that capture end-stage physical reclusion, our sample represents an at-risk digital population that is actively engaged in the remote workforce. The fact that we identified significant negative associations with social and challenge traits in a population hovering at the cutoff suggests that digital behavioral shifts occur early in the marginalization process, before total physical withdrawal is achieved. However, this high concentration of threshold-risk individuals means our results should be generalized to at-risk gamer populations rather than the general Japanese public. Consequently, while our findings establish a roadmap for early-stage intervention, future research with clinical populations is needed to assess how these preferences evolve as players move further along to more extreme stages of the social withdrawal spectrum.

Furthermore, the CFA conducted on the HEXAD and GTS did not demonstrate acceptable construct validity on the full scales. Future studies could benefit from validating these scales in the Japanese language before investigating Japanese gaming preferences and user motivations. Nevertheless, the CFA did show acceptable construct validity for our Hypothesised variables, so we maintain the validity of this study’s main findings.

The cross-sectional nature of the data only allows us to glimpse participants’ current gaming preferences and psychological risk levels. Consequently, these results must be interpreted as strictly associative rather than causal. While significant links between social marginalization risk and specific player traits were identified through this analysis, it remains unclear whether these psychological dispositions and preferences are a precursor to, or a result of, varying degrees of social withdrawal. To avoid misleading interpretations and applications of these relationships, future research should focus on longitudinal analyses to investigate how gaming preferences may change dynamically over time alongside one’s social condition.

A further limitation of this study is its exclusive reliance on self-report measures, which may be subject to reporting bias or social desirability, particularly in sensitive domains related to social withdrawal and impairment. The psychometric tools used, such as the NHR [3] and HEXAD [29,30] scales, are validated for this population, but they do not capture real-time player behavior. Future research should investigate these psychometric findings using objective behavioral data, such as in-game activity logs, the frequency of multiplayer participation, and digital communication patterns between players. Incorporating such metrics could provide a more robust understanding of how the psychological dispositions identified in this study translate into actual engagement within virtual environments.

The practical value of this study’s trait-based approach lies in its ability to derive genre-independent design requirements that are robust across various virtual environments. By identifying stable psychological dispositions —-such as the observed aversion to competition and high-difficulty tasks—- we can propose specific mechanics, like cooperative-only social loops and low-friction, RPG-like progression, that can be implemented regardless of whether the game ‘vessel’ is an Action game or a Visual Novel, for example. These findings provide a strong, theory-driven foundation for needs-based design grounded in Self-Determination Theory. [80] However, a limitation of the current study is the lack of granularity regarding specific, day-to-day gaming behaviors, such as a detailed breakdown of time spent in multiplayer versus single-player modes or the frequency of specific types of social interaction. While our trait-focused categories provide a stable roadmap, the absence of this behavioral data may impede immediate title-specific translational impact. Future research should therefore aim to capture these play patterns in more detail, refining the general psychological profiles into precise, highly specific design recommendations.

Finally, while the NHR Scale is strongly correlated with social withdrawal, it is important to note that the criteria for a formal hikikomori diagnosis are significantly more stringent. Past research illustrates this gap: while 22.3% of a sample might be classified as ’High NHR,’ only 1.4% typically exhibit the severe behaviors required for a clinical diagnosis [62]. Our study specifically focuses on general gaming preferences associated with social marginalization risk, capturing psychological and behavioral orientations that may exist independently of a formal diagnosis. Because our focus was limited to this broader isolation risk, we did not examine comorbid conditions like autism, which can share psychiatric traits with severe withdrawal [81]. Similarly, we did not examine participants’ potential depression and social anxiety levels, which have been reported in high levels in previous hikikomori studies [12]. Thus, we did not investigate how these comorbidities might further influence gaming preferences. Future research should incorporate clinical samples and investigate a wider range of psychiatric conditions to better understand how formal diagnoses, such as hikikomori, specifically relate to game traits and player motivations.

## 8. Conclusion

This study sheds light on the relationships among social marginalization risk, gamer traits, and the motivations of Japanese players. It reflects their inclinations and preferences in consuming and enjoying video games. The findings show a negative relationship between participants’ NEET-Hikikomori risk tendencies and traits related to helping other players in games (H1), as well as constructs related to social interaction and connection in games (H2a, H3a). These results remained robust even after controlling for age, gender, and extraversion. Our exploratory analyses also revealed negative relationships between NHR level and constructs related to in-game challenges, free expression of creativity, and player motivation via extrinsic rewards.

These results suggest that social withdrawal predispositions seen in high-risk players is not merely present in physical, real-world environments, but can also extend to virtual environments. This means that standard social mechanics could be unappealing or even stressful for some high-risk people. Furthermore, the observed aversion towards achievement, challenges, and creative self-expression implies that traditional game-based rewards and great difficulty tasks could lead to disengagement, rather than motivation, for this specific demographic. Therefore, for videogames to serve as an “entryway” for resocialisation, designers should take care to develop safe, low-pressure environments that can accommodate the inhibited self-expression and specific motivational orientations preferred by socially marginalized players.

In summary, we demonstrated that players with a higher risk of social marginalization exhibit lower preferences towards socialising, achievement, and challenge-related player traits. Future studies could explore other potential associations between such tendencies and established psychopathological propensities, such as depression, autism, hikikomori, and anxiety risk, as well as research possible causal links.
