# Supplementary material for: Social marginalization risk and its negative association with socialising preferences in Japanese gamers
Source: PLoS One. 2026 Jul 9;21(7):e0353122. doi: 10.1371/journal.pone.0353122 (PMC13349300; doi:10.1371/journal.pone.0353122)
Supplement: Attachment — Submitted filename: Response to Reviewers - Plos Mental ONE.docx [file pone.0353122.s002.docx]

**For editorial board and reviewers:**

We thank you for your careful consideration of our manuscript “Social marginalization risk and its negative association with socialising preferences in Japanese gamers“ and your useful comments. In this letter, we provide point-by-point responses to those comments, addressing questions and problems pointed out by the reviewers. The original reviewer comments will be marked in **bold**, while the responses will be marked in blue. Additions included directly in the manuscript will be marked in red here.

--------------------------------------------------------------------------------------------------------------------

**Reviewer #1:**

**1. Sample characterization and recruitment**

**The inclusion and exclusion criteria should be described in greater detail. Given the inherent difficulty in accessing individuals with high levels of social withdrawal, the authors should further elaborate on their recruitment strategy and discuss whether it adequately captures individuals at the more severe end of the NEET/hikikomori spectrum. There is a potential risk of selection bias, particularly if participants were recruited through online platforms that may exclude the most socially withdrawn individuals.**

Thank you for your comment. The inclusion and exclusion criteria were already detailed in the Participants section, but were rewritten for clarity as follows:

All participants were 18 or older, and each received 200 JPY for completing the survey. The survey was open to people with Japanese nationality and residence who were 18 or older and had previous experience with video games.

Exclusion criteria included participation in both survey rounds, failing at least one of two attention checks, leaving before fully completing the questionnaire, and not being born and raised in Japan.

Of our original 676 participants, 89 had invalid responses according to our exclusion criteria, and were thus excluded from the analysis.

Convenience sampling was conducted via Crowdworks because hikikomori and those at high risk of social isolation are traditionally avoidant of social-facing employment and are likely to seek remote, anonymous freelance work. This makes online sampling ideal for our target audience, as it potentially targets a larger population of hikikomori across the hikikomori risk spectrum. (For more information on the advantages of convenience sampling, please refer to page 19 of Landers, R. N., & Behrend, T. S. (2015). An inconvenient truth: Arbitrary distinctions between organizational, Mechanical Turk, and other convenience samples. Industrial and Organizational Psychology, 8(2), 142-164.
While recruitment was conducted via convenience sampling, the study maintained high internal validity by utilizing the validated NHR Scale as a psychometric filter to identify high-risk individuals, ensuring that the sample was representative of hikikomori profiles in Japan. This was added to the “Participants” sub-section of our Methods:

To address the potential bias of convenience sampling and ensure the quality of our data, we implemented the validated NEET-Hikikomori Risk (NHR) Scale to measure the hikikomori risk of participants. We found that results ranged between 44 and 153 points, from mild to more extreme risk of social isolation.

While recruitment was conducted via convenience sampling, the use of the validated NHR Scale allowed us to measure the risk levels within this specific digital demographic. Our sample mean (M=103.4) sits just below the proposed 104-point cutoff for ‘High Risk' (Uchida & Norasakkunkit, 2015), resulting in a distribution where 52.3% of participants are classified as high-risk. This distribution is consistent with our recruitment strategy, which specifically targeted individuals with gaming experience on a crowdsourcing platform —- two factors known to correlate with the NEET-hikikomori lifestyle (Stavropoulos et al., 2019). Consequently, our sample aligns with contemporary research showing that the hikikomori phenomenon increasingly affects a diverse, middle-aged digital workforce across genders (Yong et al., 2020).

Because our research goals focus on understanding the relationship between specific gaming preferences and the risk of social isolation, rather than estimating nationwide prevalence, the use of the validated NHR scale provides us the necessary tools for our comparative analysis without the requirement for population-wide weighting.

**2. Clinical variables and potential confounders**

**Hikikomori is frequently associated with psychiatric conditions such as depression, social anxiety, and autism spectrum traits. The absence of these variables in the data collection is a notable limitation, as they may act as important confounders or mediators. The authors should justify this omission and explicitly acknowledge it in the discussion.**

We appreciate this important point. We did not collect measures of associated psychiatric conditions. Besides the already mentioned autism spectrum traits in our current Limitations section, we have appended the following section to explicitly state the lack of measurements for the other mentioned conditions:

Because our focus was limited to this broader isolation risk, we did not examine comorbid conditions like autism, which can share psychiatric traits with severe withdrawal (*Dell’Osso et al., 2023*). Similarly, we did not examine participants' potential depression and social anxiety levels, which have been reported in high levels in previous hikikomori studies (*Koyama et al., 2010*). Thus, we did not investigate how these comorbidities might further influence gaming preferences. Future research should incorporate clinical samples and investigate a wider range of psychiatric conditions to better understand how formal diagnoses, such as hikikomori, specifically relate to game traits and player motivations.

**3. Methodological transparency**

**Additional methodological details would improve reproducibility. Specifically:**

**Which statistical software and packages were used for data analysis?**

**How were missing data handled, particularly for incomplete questionnaire responses?**

**What was the average time required to complete the survey?**

Thank you for your comment. The details on the software were included at the beginning of the Methods section:

All materials, data, and analyses were done with R for data formatting and analysis (version 2023.03.1+446); we utilized the ‘readxl' package for library import, ‘tidyverse' for plotting, ‘moments' to calculate skewness and kurtosis, ‘lm.beta' to calculate standardized β coefficients, ‘effectsize' to interpret the R² results, and ‘xtable' and ‘knitr' for table building. The source code and analysis …

Missing data and average completion time were mentioned in the Participants sub-section:

Exclusion criteria included having answered the questionnaire more than once (in the same round or across both survey rounds), failing at least one of two attention checks, leaving before fully completing the questionnaire, and not being born and raised in Japan.

[…]

The average completion time of the questionnaire was around 32 minutes.

**4. Reporting of results**

**The abstract would benefit from a clearer presentation of the statistical significance and direction of the main findings. This would improve accessibility and ensure that key results are immediately interpretable.**

Thank you for your comment. We have complemented the abstract to clearly state the connections between the relevant constructs and their interpretations:

After accounting for age, gender and extraversion, results showed that higher NHR score was associated with lower HEXAD Socialiser, lower HEXAD Philanthropist and lower Game Traits Social orientation scores, suggesting a negative association between NEET-Hikikomori risk and player traits related to socialisation. Furthermore, exploratory analysis showed that a higher NHR score was associated with lower scores in HEXAD Achiever and Game Traits Challenges orientation, suggesting NEET-Hikikomori risk has a negative correlation with challenges and achievement-related gameplay.

Furthermore, we have clarified this in the “Hypothesised analyses” sub-section to facilitate the understanding of the direction of our findings throughout the manuscript:

In other words, **higher NHR score was associated with lower HEXAD Socialiser, lower HEXAD Philanthropist, and lower GTS Social Orientation scores**.

A similar summary sentence was also included in the “Exploratory analysis” sub-section:

In other words, a **higher NHR score was associated with lower scores in the HEXAD Free Spirit, HEXAD Achiever, HEXAD Player, Game Traits Narrative, and Game Traits Challenge constructs, even after accounting for age and gender.**

**5. Interpretation of findings**

**The conclusion that individuals at higher withdrawal risk are less socially engaged even in gaming environments may be somewhat unidimensional. Alternative interpretations should be considered. For example, a preference for solitary or low-pressure forms of engagement may represent a coping strategy rather than a deficit. Expanding the discussion to include this perspective would add important nuance.**

Thank you for your comment. As the reviewer points out, there may be more reasons that could lead those at higher risk of social isolation to want to avoid social interactions in gaming environments. This has been reflected in the Discussion section:

It is important to consider, however, that this observed preference for lower social engagement should not be interpreted solely as a psychological deficit. Instead, such tendencies may represent a proactive coping strategy used by high-risk players to manage their emotional well-being in digital environments, particularly those that can lead to stressful situations. Previous research *(Frommel, Johnson & Mandryk, 2023)* found that “players who perceived their gaming communities as toxic also reported higher loneliness and lower need satisfaction of relatedness”. Considering these results, gravitating toward more solitary forms of engagement could be the way some players avoid the `vicious cycle' of toxicity found in some online gaming communities. One possible medium that could offer 'low-risk' experiences are narrative-driven Visual Novels, which remove the immediate pressure of direct social confrontation and the stress of negative feedback, while still offering the possibility of forming parasocial relationships with the characters in the stories. This allows players to feel like they are maintaining their ‘digital social capital' *(Ellison, Steinfield, & Lampe, 2007)* while protecting themselves from further social impairment.

**6. Reliance on self-report measures**

**All variables were assessed via self-report, which introduces the possibility of reporting bias, particularly in sensitive domains such as social functioning. The authors should acknowledge this limitation more explicitly and consider the value of incorporating behavioral data (e.g., in-game activity logs, multiplayer participation, communication patterns) in future research.**

Thank you for this insightful point. We understand that the exclusive use of self-report measures could be a bias-inducing limitation. However, given the reclusive nature of the high-risk NEET-hikikomori population, anonymous online surveys are currently one of the most effective methods to reach these individuals, as they often seek remote and anonymous environments. To mitigate this issue, we decided to utilize the NHR scale, which has been externally validated and shown to strongly correlate with actual behavioral indicators of social isolation. Nevertheless, we have updated our Limitations section to explicitly address this bias and propose the integration of behavioral data in future research.

A further limitation of this study is its exclusive reliance on self-report measures, which may be subject to reporting bias or social desirability, particularly in sensitive domains related to social withdrawal and impairment. The psychometric tools used, such as the NHR (Uchida & Norasakkunkit, 2015) and HEXAD (*Diamond et al., 2015; Tondello et al., 2016*) scales are validated for this population, but they do not capture real-time player behavior. Future research should investigate these psychometric findings using objective behavioral data, such as in-game activity logs, the frequency of multiplayer participation, and digital communication patterns between players. Incorporating such metrics could provide a more robust understanding of how the psychological dispositions identified in this study translate into actual engagement within virtual environments.

**7. Granularity of gaming variables**

**The study would benefit from greater specificity regarding gaming behaviors and preferences (e.g., game genres, multiplayer versus single-player engagement, types of social interaction). This limitation should be acknowledged, as it constrains the practical implications of the findings.**

Thank you for your comment. We agree that providing greater specificity regarding gaming behaviors, such as the balance of multiplayer versus single-player engagement and more specific game genres, would enrich the findings of this study. However, the primary aim of this research was to move beyond broad game genres, to instead identify the **underlying psychological traits and motivational orientations** of high-risk players. By focusing on stable traits (through measurements via the HEXAD and GT Scales), we can provide a more flexible theoretical foundation, which could be applied across various genres. For instance, our main finding that high-risk gamers have lower “Social” and “Challenge” orientations allows us to propose **genre-independent design requirements**, such as shifting focus from competition to cooperation, or implementing simple progression mechanics to avoid sudden shifts in the challenge level. We have updated the Limitations section to address this lack of granularity and discuss its implications for the study’s practical limitations:

The practical value of this study’s trait-based approach lies in its ability to derive genre-independent design requirements that are robust across various virtual environments. By identifying stable psychological dispositions —-such as the observed aversion to competition and high-difficulty tasks—- we can propose specific mechanics, like cooperative-only social loops and low-friction, RPG-like progression, that can be implemented regardless of whether the game `vessel' is an Action game or a Visual Novel, for example. These findings provide a strong, theory-driven foundation for needs-based design grounded in Self-Determination Theory *(Ryan & Deci, 2000)*.

However, a limitation of the current study is the lack of granularity regarding specific, day-to-day gaming behaviors, such as a detailed breakdown of time spent in multiplayer versus single-player modes or the frequency of specific types of social interaction. While our trait-focused categories provide a stable roadmap, the absence of this behavioral data may impede immediate title-specific translational impact. Future research should therefore aim to capture these play patterns in more detail, refining the general psychological profiles into precise, highly specific design recommendations.

Furthermore, the practical value of this trait-based approach is shown by our ability to derive genre-independent design requirements, which can be seen in the following question.

**8. Implications for intervention**

**While the manuscript highlights the relevance of tailoring game design to different player profiles, the implications for intervention remain somewhat underdeveloped. More concrete, theory-driven suggestions would strengthen the translational impact of the study.**

Thank you for your suggestion. To strengthen the translational impact of our study, we have expanded the Discussion section to include the “Design suggestions and implications for interventions” sub-section. In it, we suggest concrete, theory-driven design implications grounded in the Self-Determination Theory (SDT) *(Ryan & Deci, 2000)*. By mapping our trait-based findings to the SDT’s core needs for Competence, Autonomy and Relatedness, we propose that interventions for high-risk players could: (1) prioritize cooperative social mechanics over competition, to bypass social anxiety and fears of toxicity (which could help with players’ Relatedness needs); (2) utilize steady, RPG-like progression and 'simple mechanics' to support players’ Competence needs, without the risk of disengagement from negative feedback; and (3) implement short, manageable play sessions to support players’ Autonomy needs while avoiding physical and mental fatigue. These additions provide a clearer roadmap for developers to move from general psychometric profiles to functional game design.

To move from psychometric profiles and traits explored in this study to more practical interventions, our findings can be interpreted into more grounded game design suggestions, using the **Self-Determination Theory** (SDT) as a foundation for our suggestions *(Ryan & Deci, 2000)*.

The SDT is a macro-theory of human motivation which posits that for individuals to experience high-quality engagement and well-being, three basic psychological needs must be satisfied: autonomy (the feeling of agency and choice), competence (the experience of mastery and effectiveness), and relatedness (the sense of connection and belonging with others) *(Ryan & Deci, 2000).*

Thus, we posit that the game design elements we suggest should fulfill players' three core needs, as described by the SDT. For individuals with high marginalization risk, who seem to show an aversion towards traditional challenges and social situations that may create social pressure, we propose that the game design should focus on:

- **Steady, RPG-like progression and 'simple mechanics’**: Mechanics should be slowly introduced to avoid sharp increases in difficulty. This allows players to experience a sense of growth and mastery without the difficult hurdles and negative feedback that frequently lead to disengagement in this population, and thus can foster their Competence needs.
- **Focus on cooperative gameplay rather than competitive:** This can help with players' Relatedness needs, by allowing players to interact with one another, while mitigating the fears of being a 'nuisance' or facing toxic interactions, which currently act as a barrier to social participation for high-risk players. Similar to the RPG-like mechanics, social elements should also aim for a slow-start entry point, being introduced slowly to avoid any sudden negative feedback, and progress towards long-term interaction.
- **Short, flexible play sessions:** This provides players with the agency to manage their own visual or physical fatigue, ensuring the digital environment remains an inviting ‘gate' rather than a source of stress. This helps to avoid a buildup of stress over long play sessions, while also fostering players' Autonomy needs.

--------------------------------------------------------------------------------------------------------------------

**Reviewer #2:**

**Major Concerns**

**1. The manuscript states that data were collected “in two rounds over a span of two weeks” , but it is unclear what the purpose of this design was. Were there any differences in measurement, manipulation, or quality control across rounds? If not, the rationale for this design should be clarified.**

Thank you for your comment. The execution of a second round was to remove an English translation of the questionnaire that accompanied the original Japanese questions, as it was decided that it was not contextually relevant, given that the vast majority of our eligible participants would be native Japanese speakers, and thus would not need this translation. The content and order of the Japanese questions were not altered in any way. This change was done on the second day of the survey’s release, and therefore, most of the answers collected happened in the version without translation. This was reflected in the Participants section:

The first round, which lasted for a day, contained the original Japanese text and a translation into English language. However, considering that most eligible participants would not need this translation, it was decided that the translation was not contextually relevant, and thus removed it in the second round, which was available for new participants for the following 13 days.

**2. The mean NHR score (M = 103.4) is extremely close to the proposed cutoff (104), and more than half of the sample is classified as “high risk”. This distribution is somewhat counterintuitive given prior epidemiological evidence suggesting much lower prevalence rates. While the authors attribute this to recruitment via a crowdsourcing platform, the implications for sampling bias and generalizability need more explicit discussion. In particular, how this sample differs from community or clinical populations should be clearly articulated.**

We thank the reviewer for this relevant observation.

We have expanded our Participants section to explain the use of our convenience sampling, and how it helps our results align with contemporary research on the hikikomori phenomenon:

While recruitment was conducted via convenience sampling, the use of the validated NHR Scale allowed us to measure the risk levels within this specific digital demographic. Our sample mean (M=103.4) sits just below the proposed 104-point cutoff for 'High Risk' (Uchida & Norasakkunkit, 2015), resulting in a distribution where 52.3% of participants are classified as high-risk. This distribution is consistent with our recruitment strategy, which specifically targeted individuals with gaming experience on a crowdsourcing platform —- two factors known to correlate with the NEET-hikikomori lifestyle (Stavropoulos et al., 2019). Consequently, our sample aligns with contemporary research showing that the hikikomori phenomenon increasingly affects a diverse, middle-aged digital workforce across genders (Yong et al., 2020).

We have also expanded our Limitations section to explicitly discuss the implications of this distribution and directions future research could take.

The ‘borderline' nature of our sample mean (M=103.4) compared to the 104-point cutoff from the literature (Uchida & Norasakkunkit, 2015) provides a unique opportunity to investigate the threshold of social withdrawal risk. Unlike clinical samples that capture end-stage physical reclusion, our sample represents an at-risk digital population that is actively engaged in the remote workforce. The fact that we identified significant negative associations with social and challenge traits in a population hovering at the cutoff suggests that digital behavioral shifts occur early in the marginalization process, before total physical withdrawal is achieved. However, this high concentration of threshold-risk individuals means our results should be generalized to at-risk gamer populations rather than the general Japanese public. Consequently, while our findings establish a roadmap for early-stage intervention, future research with clinical populations is needed to assess how these preferences evolve as players move further along to more extreme stages of the social withdrawal spectrum.

We argue that our sample represents a specific at-risk digital demographic (gamers and crowdsourced workers), rather than a general community sample. By capturing a population that sits at the psychological threshold of withdrawal, our study identifies how social and challenge orientations in games begin to shift even before an individual reaches the severe clinical criteria for hikikomori. We believe this 'threshold' focus is vital for identifying early-intervention opportunities through game design. We have also clarified that these findings are intended for generalization to at-risk digital populations rather than the general population.

**3. The reporting of measurement properties is insufficiently systematic. While some reliability estimates are provided (e.g., Cronbach’s α for selected subscales), a complete report of internal consistency for all scales and subdimensions—especially those directly used in hypothesis testing—is necessary. Similarly, in the Construct Validity section, it is unclear: 1) What exact CFA models were specified; 2) Why only partial models (e.g., selected subdimensions) were evaluated rather than full theoretical structures for each instrument (NHR, HEXAD, GTS, TIPI-J). Even if some models demonstrate poor fit, these results should be transparently reported and explicitly addressed in the limitations.**

Thank you for your comment. For the sake of clarity, we believe that providing the reliability and consistency estimates for the variables relevant to our main hypotheses would be the best approach. As the remaining variables would be beyond the scope of our research, we did not believe it to be necessary to provide scores for all variables outside our hypotheses.

This is particularly relevant given the poor validity of the HEXAD and GTS constructs when all subscales are included. However, as most of these subscales are not relevant to our hypotheses, we believe that focusing on the relevant subscales would be sufficient. Furthermore, the main reason we only reported construct validity for HEXAD and GTS scales was because we conducted the translation and back-translation process ourselves; the TIPI-J (Oshio, Shingo & Cutrone, 2012) and NHR (Uchida & Norasakkunkit, 2015) scales have been previously validated in a Japanese sample and published, and so their respective construct validity can be found in their respective publications. The HEXAD and GTS scales, however, have not been previously validated, therefore our translated versions needed additional validation with our Japanese sample.

**4. In Section 5.1.1, only selected correlations are reported. A full correlation matrix among all key variables would substantially improve transparency and interpretability. This is particularly important given the conceptual overlap between constructs (e.g., HEXAD Socialiser and GTS Social Orientation) and would also be beneficial for further meta-analysis by the other researchers.**

Thank you for your comment. The full correlation between all remaining variables is currently provided in Table 6 and Table 7.

**5. The statement that associations remain significant after adding covariates and therefore are “not explained” by them (p.12, lines 410–412) is conceptually inaccurate. Covariates (e.g., age, gender, extraversion) are included precisely because they are theoretically related to both predictors and outcomes (I’d like to appreciate the authors efforts in this point, since this is an often-overlooked part in some empirical studies). A more appropriate interpretation would be that the associations remain statistically significant after accounting for these covariates.**

Thank you for your suggestion and the positive feedback. We agree that the term used should be adjusted to better reflect the findings. We have revised the manuscript to state that the observed associations remain statistically significant after accounting for the influence of the covariates:

These results were robust even after including age, gender, and extraversion as covariates, demonstrating that the negative associations remained statistically significant after accounting for demographics and introverted personality dispositions in our Japanese sample.

**6. On p.12 (around line 430), the manuscript argues that a negative association is supported by prior work showing a positive association between social support and HEXAD Socialiser. The logical link here is not sufficiently articulated. The authors should clarify the conceptual bridge (e.g., whether social support is assumed to be inversely related to marginalization risk).**

Thank you for your suggestion. The logical link the reviewer points out rests on the empirical finding that high NEET-Hikikomori Risk (NHR) is characterized by a lack of social resources. As noted by Uchida & Norasakkunkit (2015), NHR scores are strongly and negatively correlated with close relationships and general well-being.

Furthermore, the definition of the HEXAD Philanthropist and Socialiser constructs establish their positive association with Extraversion *(Tondello et al., 2016)*. Fong et al. (2024) reiterated such findings, showing that social support is a positive predictor of HEXAD Socialiser and Philanthropist traits. Similarly, in the Game Traits Scale also used in this study, the Social orientation is also positively correlated with Extraversion *(Tondello et al., 2019)*

Given the above, it follows logically that a risk scale measuring the **absence** of such social support (NHR) would show a negative correlation to the three HEXAD and Game Traits constructs cited above. We have updated our Discussion to make this inverse relationship between marginalization risk and social support more explicit:

The moderate negative association between the NHR Score and the HEXAD Philanthropist, HEXAD Socialiser and Game Traits Social scores all suggest an inverse relationship between preferences towards social-based gameplay and social marginalization risk. This is consistent with the definitions for the HEXAD Philanthropist and Socialiser constructs and the Game Traits Social orientation, all of which are negatively associated with Extraversion. Furthermore, the HEXAD Scale validation made by Fong et al. further corroborated that social support was positively associated with the Philanthropist construct.

This comparison is grounded in the fact that high NHR scores are fundamentally characterized by a deficiency in social resources and an inverse relationship with close social ties. Thus, the negative association identified in our study aligns with the positive association found in prior work: while social support encourages prosocial traits, the risk of marginalization appears to inhibit them.

**7. Although the manuscript generally maintains an associative tone, the cross-sectional nature of the data should be more explicitly acknowledged as a limitation. This can effectively avoid misleading interpretations made by the publics or medias.**

Thank you for your comment. We agree that the cross-sectional nature of the study should be clearly expressed and improvements should be made in future studies. We have adjusted the Limitations section to more clearly explain this point:

The cross-sectional nature of the data only allows us to glimpse participants’ current gaming preferences and psychological risk levels. Consequently, these results must be interpreted as strictly associative rather than causal. While significant links between social marginalization risk and specific player traits were identified through this analysis, it remains unclear whether these psychological dispositions and preferences are a precursor to, or a result of, varying degrees of social withdrawal. To avoid misleading interpretations and applications of these relationships, future research should focus on longitudinal analyses to investigate how gaming preferences may change dynamically over time alongside one's social condition.

**Minor Concerns**

**1. The OSF view-only link is not accessible. Please verify the URL prior to publication.**

Thank you for your feedback. The URL itself was in fact correct, but due to a problem with the Latex formatting, it was not redirecting to the correct website upon click. This has been corrected and the link to access the OSF repository is copied below.

<https://osf.io/wp2rh/?view_only=0c2e804fc4d341b0b18801226c1ac48b>

**2. Terminology consistency: Since TIPI-J measures extraversion–introversion on a single continuum, the use of the term “Introversion” as a standalone label (p.9) may be confusing. Please standardize terminology (e.g., “Extraversion” or “Extraversion/Introversion”).**

Thank you for your comment. We have updated the manuscript to prioritize “extraversion” as our primary term, particularly when discussing our own variables and results. For consistency, when referencing research that utilized the term “introversion”, we have included the corresponding inverse equivalent (e.g. “low extraversion”) in parentheses, such as the example below:

For instance, Bonnaire and Roignot investigated the relationships between personality dimensions, coping strategies, and the hikikomori condition, and found that introversion (or low extraversion) was positively correlated with the hikikomori levels.

This ensures a consistent labeling throughout the manuscript, while accurately reflecting the original language of cited works.

**3. Typographical issue: “benchmark mode” (p.9) likely should be “benchmark model.”**

Thank you for your feedback. This has been corrected.

**4. Model naming: Terms such as “Hypothesis variable 1/2” are unnecessary and potentially confusing. Standard labels such as Model 1, Model 2, etc., would improve clarity.**

We thank the reviewer for this suggestion. We understand that standard labels such as 'Model 1' and 'Model 2' are preferred for scientific consistency. However, given the number of variables and the distinction between our primary hypotheses and exploratory analyses, we were concerned that generic labels alone might require readers to navigate away from the tables to recall specific model parameters.

To address both concerns, we have updated the naming convention to a standardized format (Model 1, Model 2, etc.) while retaining detailed descriptors in the table titles and captions. This ensures that the models follow a conventional sequence while remaining 'atomically' understandable. We have updated Tables 3 through 7 accordingly, and added some mentions in the text to these changes, as below:

In Model 1, we first executed three separate regression models to investigate possible associations between the NHR Score…

Among the variables in Model 4, the HEXAD Free Spirit, HEXAD Player, and GTS Challenge factors showed significant negative relationships with the NHR Score, marked by weak effect sizes.

**5. On p.12, the reference to “the Chinese 12-item version of the HEXAD scale” is confusing given that the present study is conducted in Japan. This should be clarified or revised.**

Thank you for your comment. The cited study pertains to the same scale, and although it was being validated in a different language and conducted in another country, the results align with the findings in our Japanese sample. We adjusted the wording to make the difference between the studies clearer.

This finding is consistent with results from previous research conducted in Hong Kong (Fong et al., 2024), which showed that social support was positively associated with the Philanthropist construct during the validation of the Chinese 12-item version of the HEXAD Scale. This suggests that philanthropic orientations in games may be less interesting for people with higher risk of social marginalization. However, further confirmatory testing would be needed to determine whether these psychological associations remain stable across different regional contexts.

**References**

Dell’Osso, L., Amatori, G., Muti, D., Giovannoni, F., Parri, F., Violi, M., ... & Carpita, B. (2023). Autism spectrum, hikikomori syndrome and internet gaming disorder: Is there a link?. Brain Sciences, 13(7), 1116.

Diamond, L., Tondello, G. F., Marczewski, A., Nacke, L. E., & Tscheligi, M. (2015). The HEXAD gamification user types questionnaire: Background and development process. In CHI PLAY 2015-The ACM SIGCHI Annual Symposium on Computer-Human Interaction in Play

Ellison, N. B., Steinfield, C., & Lampe, C. (2007). The benefits of Facebook “friends:” Social capital and college students’ use of online social network sites. Journal of computer‐mediated communication, 12(4), 1143-1168.

Fong, T. C., Lu, S., Ye, X., Junus, A., & Yip, P. S. (2025). Validation of the 12-item gamification user types hexad scale in 1,610 Hong Kong young gamers. International Journal of Mental Health and Addiction, 23(6), 4531-4549.

Frommel, J., Johnson, D., & Mandryk, R. L. (2023). How perceived toxicity of gaming communities is associated with social capital, satisfaction of relatedness, and loneliness. Computers in Human Behavior Reports, 10, 100302.

Koyama, A., Miyake, Y., Kawakami, N., Tsuchiya, M., Tachimori, H., Takeshima, T., & World Mental Health Japan Survey Group. (2010). Lifetime prevalence, psychiatric comorbidity and demographic correlates of “hikikomori” in a community population in Japan. Psychiatry research, 176(1), 69-74.

Oshio, A., Shingo, A. B. E., & Cutrone, P. (2012). Development, reliability, and validity of the Japanese version of Ten Item Personality Inventory (TIPI-J). Japanese Journal of Personality/Pasonariti Kenkyu, 21(1).

Ryan, R. M., & Deci, E. L. (2000). Self-determination theory and the facilitation of intrinsic motivation, social development, and well-being. American psychologist, 55(1), 68.

Stavropoulos, V., Anderson, E. E., Beard, C., Latifi, M. Q., Kuss, D., & Griffiths, M. (2019). A preliminary cross-cultural study of Hikikomori and Internet Gaming Disorder: The moderating effects of game-playing time and living with parents. Addictive Behaviors Reports, 9, 100137.

Tondello, G. F., Wehbe, R. R., Diamond, L., Busch, M., Marczewski, A., & Nacke, L. E. (2016, October). The gamification user types hexad scale. In Proceedings of the 2016 annual symposium on computer-human interaction in play (pp. 229-243).

Tondello, G. F., Arrambide, K., Ribeiro, G., Cen, A. J. L., & Nacke, L. E. (2019, August). “I don’t fit into a single type”: A Trait Model and Scale of Game Playing Preferences. In Ifip conference on human-computer interaction (pp. 375-395). Cham: Springer International Publishing.

Uchida, Y., & Norasakkunkit, V. (2015). The NEET and Hikikomori spectrum: Assessing the risks and consequences of becoming culturally marginalized. Frontiers in psychology, 6, 1117.

Yong, R. K., Fujita, K., Chau, P. Y., & Sasaki, H. (2020). Characteristics of and gender difference factors of hikikomori among the working-age population: A cross-sectional population study in rural Japan. Nihon Koshu Eisei Zasshi (JAPANESE JOURNAL OF PUBLIC HEALTH), 67(4), 237-246.
