# Supplementary material for: Social marginalization risk and its negative association with socialising preferences in Japanese gamers
Source: PLoS One. 2026 Jul 9;21(7):e0353122. doi: 10.1371/journal.pone.0353122 (PMC13349300; doi:10.1371/journal.pone.0353122)
Supplement: Peer review history

# Peer review: Decision Letter 0

**DOI:** 10.1371/journal.pone.0353122.r001

**Authors:** Marc Potenza

19 Apr 2026

PONE-D-26-12060

Social marginalization risk and its negative association with socialising preferences in Japanese gamers

PLOS One

Dear Dr. Confessor,

Thank you for submitting your manuscript to PLOS ONE. After careful consideration, we feel that it has merit but does not fully meet PLOS ONE’s publication criteria as it currently stands. Therefore, we invite you to submit a revised version of the manuscript that addresses the points raised during the review process.

Please see the reviewers' comments for details and respond to the points raised in a point-by-point fashion.

Please submit your revised manuscript by Jun 03 2026 11:59PM. If you will need more time than this to complete your revisions, please reply to this message or contact the journal office at plosone@plos.org. When you're ready to submit your revision, log on to https://www.editorialmanager.com/pone/ and select the 'Submissions Needing Revision' folder to locate your manuscript file.

Please include the following items when submitting your revised manuscript:

- A letter that responds to each point raised by the academic editor and reviewer(s). You should upload this letter as a separate file labeled 'Response to Reviewers'.
- A marked-up copy of your manuscript that highlights changes made to the original version. You should upload this as a separate file labeled 'Revised Manuscript with Track Changes'.
- An unmarked version of your revised paper without tracked changes. You should upload this as a separate file labeled 'Manuscript'.

If you would like to make changes to your financial disclosure, please include your updated statement in your cover letter. Guidelines for resubmitting your figure files are available below the reviewer comments at the end of this letter.

If applicable, we recommend that you deposit your laboratory protocols in protocols.io to enhance the reproducibility of your results. Protocols.io assigns your protocol its own identifier (DOI) so that it can be cited independently in the future. For instructions see: https://journals.plos.org/plosone/s/submission-guidelines#loc-laboratory-protocols. Additionally, PLOS ONE offers an option for publishing peer-reviewed Lab Protocol articles, which describe protocols hosted on protocols.io. Read more information on sharing protocols at https://plos.org/protocols?utm_medium=editorial-email&utm_source=authorletters&utm_campaign=protocols.

As the corresponding author, your ORCID iD is verified in the submission system and will appear in the published article. PLOS supports the use of ORCID, and we encourage all coauthors to register for an ORCID iD and use it as well. Please encourage your coauthors to verify their ORCID iD within the submission system before final acceptance, as unverified ORCID iDs will not appear in the published article. *Only*  the individual author can complete the verification step; PLOS staff *cannot*  verify ORCID iDs on behalf of authors.

We look forward to receiving your revised manuscript.

Kind regards,

Marc N Potenza

Academic Editor

PLOS One

**Journal Requirements:**

When submitting your revision, we need you to address these additional requirements.

1. Please ensure that your manuscript meets PLOS ONE's style requirements, including those for file naming. The PLOS ONE style templates can be found at

https://journals.plos.org/plosone/s/file?id=wjVg/PLOSOne_formatting_sample_main_body.pdf and

https://journals.plos.org/plosone/s/file?id=ba62/PLOSOne_formatting_sample_title_authors_affiliations.pdf

2. We note that the grant information you provided in the ‘Funding Information’ and ‘Financial Disclosure’ sections do not match.

When you resubmit, please ensure that you provide the correct grant numbers for the awards you received for your study in the ‘Funding Information’ section.

3. Thank you for stating the following financial disclosure:

“Google Grant for K.L.”

Please state what role the funders took in the study.  If the funders had no role, please state: "The funders had no role in study design, data collection and analysis, decision to publish, or preparation of the manuscript."

If this statement is not correct you must amend it as needed.

Please include this amended Role of Funder statement in your cover letter; we will change the online submission form on your behalf.

4. Thank you for stating the following in the Financial Disclosure section:

“Google Grant for K.L.”

We note that you received funding from a commercial source: Google

Please provide an amended Competing Interests Statement that explicitly states this commercial funder, along with any other relevant declarations relating to employment, consultancy, patents, products in development, marketed products, etc.

Within this Competing Interests Statement, please confirm that this does not alter your adherence to all PLOS ONE policies on sharing data and materials by including the following statement: "This does not alter our adherence to PLOS ONE policies on sharing data and materials.” (as detailed online in our guide for authors http://journals.plos.org/plosone/s/competing-interests). If there are restrictions on sharing of data and/or materials, please state these. Please note that we cannot proceed with consideration of your article until this information has been declared.

Please include your amended Competing Interests Statement within your cover letter. We will change the online submission form on your behalf.

5. Please remove your figures from within your manuscript file, leaving only the individual TIFF/EPS image files, uploaded separately. These will be automatically included in the reviewers’ PDF.

6. If the reviewer comments include a recommendation to cite specific previously published works, please review and evaluate these publications to determine whether they are relevant and should be cited. There is no requirement to cite these works unless the editor has indicated otherwise.

[Note: HTML markup is below. Please do not edit.]

Reviewers' comments:

Reviewer's Responses to Questions

**Comments to the Author**

1. Is the manuscript technically sound, and do the data support the conclusions?

The manuscript must describe a technically sound piece of scientific research with data that supports the conclusions. Experiments must have been conducted rigorously, with appropriate controls, replication, and sample sizes. The conclusions must be drawn appropriately based on the data presented.

Reviewer #1: Partly

Reviewer #2: Yes

**********

2. Has the statistical analysis been performed appropriately and rigorously?

Reviewer #1: I Don't Know

Reviewer #2: Yes

**********

3. Have the authors made all data underlying the findings in their manuscript fully available?

The PLOS Data policy requires authors to make all data underlying the findings described in their manuscript fully available without restriction, with rare exception (please refer to the Data Availability Statement in the manuscript PDF file). The data should be provided as part of the manuscript or its supporting information, or deposited to a public repository. For example, in addition to summary statistics, the data points behind means, medians and variance measures should be available. If there are restrictions on publicly sharing data—e.g. participant privacy or use of data from a third party—those must be specified.

Reviewer #1: Yes

Reviewer #2: No

**********

4. Is the manuscript presented in an intelligible fashion and written in standard English?

PLOS ONE does not copyedit accepted manuscripts, so the language in submitted articles must be clear, correct, and unambiguous. Any typographical or grammatical errors should be corrected at revision, so please note any specific errors here.

Reviewer #1: Yes

Reviewer #2: Yes

**********

5. Review Comments to the Author

Please use the space provided to explain your answers to the questions above. You may also include additional comments for the author, including concerns about dual publication, research ethics, or publication ethics. (Please upload your review as an attachment if it exceeds 20,000 characters)

Reviewer #1: This study investigates whether individuals at higher risk of social withdrawal (particularly those aligned with the Japanese NEET/hikikomori profile) engage differently with the social dimensions of videogames. Using a sample of 587 participants, the authors test the hypothesis that withdrawal risk may be either positively or negatively associated with in-game socialisation and cooperation. Overall, the manuscript is clearly written and grounded in a solid theoretical and empirical background. The topic is relevant and timely, particularly given the increasing interest in digital environments as potential spaces for social engagement among vulnerable populations.

That said, several aspects of the manuscript could be strengthened:

1. Sample characterization and recruitment

The inclusion and exclusion criteria should be described in greater detail. Given the inherent difficulty in accessing individuals with high levels of social withdrawal, the authors should further elaborate on their recruitment strategy and discuss whether it adequately captures individuals at the more severe end of the NEET/hikikomori spectrum. There is a potential risk of selection bias, particularly if participants were recruited through online platforms that may exclude the most socially withdrawn individuals.

2. Clinical variables and potential confounders

Hikikomori is frequently associated with psychiatric conditions such as depression, social anxiety, and autism spectrum traits. The absence of these variables in the data collection is a notable limitation, as they may act as important confounders or mediators. The authors should justify this omission and explicitly acknowledge it in the discussion.

3. Methodological transparency

Additional methodological details would improve reproducibility. Specifically:

Which statistical software and packages were used for data analysis?

How were missing data handled, particularly for incomplete questionnaire responses?

What was the average time required to complete the survey?

4. Reporting of results

The abstract would benefit from a clearer presentation of the statistical significance and direction of the main findings. This would improve accessibility and ensure that key results are immediately interpretable.

5. Interpretation of findings

The conclusion that individuals at higher withdrawal risk are less socially engaged even in gaming environments may be somewhat unidimensional. Alternative interpretations should be considered. For example, a preference for solitary or low-pressure forms of engagement may represent a coping strategy rather than a deficit. Expanding the discussion to include this perspective would add important nuance.

6. Reliance on self-report measures

All variables were assessed via self-report, which introduces the possibility of reporting bias, particularly in sensitive domains such as social functioning. The authors should acknowledge this limitation more explicitly and consider the value of incorporating behavioral data (e.g., in-game activity logs, multiplayer participation, communication patterns) in future research.

7. Granularity of gaming variables

The study would benefit from greater specificity regarding gaming behaviors and preferences (e.g., game genres, multiplayer versus single-player engagement, types of social interaction). This limitation should be acknowledged, as it constrains the practical implications of the findings.

8. Implications for intervention

While the manuscript highlights the relevance of tailoring game design to different player profiles, the implications for intervention remain somewhat underdeveloped. More concrete, theory-driven suggestions would strengthen the translational impact of the study.

Conclusion

In summary, this is a well-conducted and relevant study that contributes to the understanding of gaming behavior among individuals at risk of social withdrawal. Addressing the points above (particularly regarding sample characterization, clinical confounders, and interpretative nuance) would further enhance the clarity, rigor, and impact of the manuscript.

Reviewer #2: I appreciate the opportunity to review this manuscript (PONE-D-26-12060). The topic is both timely and theoretically meaningful. In particular, the focus on social marginalization (e.g., Hikikomori/NEET risk) and its association with gaming-related socialization preferences addresses an important issue with clear societal and practical relevance, especially in East and Southeast Asian contexts where social withdrawal has become an increasingly visible concern. The study offers a valuable contribution by linking psychological risk factors with digital behavioral tendencies, which has implications for designing game-based or technology-mediated interventions. Despite being based on a relatively modest, cross-sectional online sample, the manuscript provides informative preliminary evidence on the associations between NEET-Hikikomori risk and gaming preferences. These findings serve as a useful empirical starting point for future longitudinal or experimental research in this domain. In this sense, I view the study as a well-positioned pilot investigation.

I would also like to highlight the strengths of the Introduction. The authors provide a comprehensive and well-structured synthesis of prior literature and, importantly, articulate competing hypotheses regarding the direction of associations between social marginalization and in-game socialization. This significantly strengthens the scientific value of the study by framing it within a theoretically grounded, hypothesis-driven approach.

That said, there remain several issues that should be addressed before the manuscript is suitable for publication. These concerns are mostly technical and conceptual clarifications, and I am confident that the authors can resolve them.

Major Concerns

1. The manuscript states that data were collected “in two rounds over a span of two weeks” , but it is unclear what the purpose of this design was. Were there any differences in measurement, manipulation, or quality control across rounds? If not, the rationale for this design should be clarified.

2. The mean NHR score (M = 103.4) is extremely close to the proposed cutoff (104), and more than half of the sample is classified as “high risk”. This distribution is somewhat counterintuitive given prior epidemiological evidence suggesting much lower prevalence rates. While the authors attribute this to recruitment via a crowdsourcing platform, the implications for sampling bias and generalizability need more explicit discussion. In particular, how this sample differs from community or clinical populations should be clearly articulated.

3. The reporting of measurement properties is insufficiently systematic. While some reliability estimates are provided (e.g., Cronbach’s α for selected subscales), a complete report of internal consistency for all scales and subdimensions—especially those directly used in hypothesis testing—is necessary. Similarly, in the Construct Validity section, it is unclear: 1) What exact CFA models were specified; 2) Why only partial models (e.g., selected subdimensions) were evaluated rather than full theoretical structures for each instrument (NHR, HEXAD, GTS, TIPI-J). Even if some models demonstrate poor fit, these results should be transparently reported and explicitly addressed in the limitations.

4. In Section 5.1.1, only selected correlations are reported. A full correlation matrix among all key variables would substantially improve transparency and interpretability. This is particularly important given the conceptual overlap between constructs (e.g., HEXAD Socialiser and GTS Social Orientation) and would also beneficial for further meta-analysis by the other researchers.

5. The statement that associations remain significant after adding covariates and therefore are “not explained” by them (p.12, lines 410–412) is conceptually inaccurate. Covariates (e.g., age, gender, extraversion) are included precisely because they are theoretically related to both predictors and outcomes (I’d like to appreciate the authors efforts in this point, since this is an often-overlooked part in some empirical studies). A more appropriate interpretation would be that the associations remain statistically significant after accounting for these covariates.

6. On p.12 (around line 430), the manuscript argues that a negative association is supported by prior work showing a positive association between social support and HEXAD Socialiser. The logical link here is not sufficiently articulated. The authors should clarify the conceptual bridge (e.g., whether social support is assumed to be inversely related to marginalization risk).

7. Although the manuscript generally maintains an associative tone, the cross-sectional nature of the data should be more explicitly acknowledged as a limitation. This can effectively avoid misleading interpretations made by the publics or medias.

Minor Concerns

1. The OSF view-only link is not accessible. Please verify the URL prior to publication.

2. Terminology consistency: Since TIPI-J measures extraversion–introversion on a single continuum, the use of the term “Introversion” as a standalone label (p.9) may be confusing. Please standardize terminology (e.g., “Extraversion” or “Extraversion/Introversion”).

3. Typographical issue: “benchmark mode” (p.9) likely should be “benchmark model.”

4. Model naming: Terms such as “Hypothesis variable 1/2” are unnecessary and potentially confusing. Standard labels such as Model 1, Model 2, etc., would improve clarity.

5. On p.12, the reference to “the Chinese 12-item version of the HEXAD scale” is confusing given that the present study is conducted in Japan. This should be clarified or revised.

**********

6. PLOS authors have the option to publish the peer review history of their article (what does this mean?). If published, this will include your full peer review and any attached files.

If you choose “no”, your identity will remain anonymous but your review may still be made public.

**Do you want your identity to be public for this peer review?**  For information about this choice, including consent withdrawal, please see our Privacy Policy.

Reviewer #1: No

Reviewer #2: **Yes:** Kunru Song

**********

[NOTE: If reviewer comments were submitted as an attachment file, they will be attached to this email and accessible via the submission site. Please log into your account, locate the manuscript record, and check for the action link "View Attachments". If this link does not appear, there are no attachment files.]

To ensure your figures meet our technical requirements, please review our figure guidelines: https://journals.plos.org/plosone/s/figures

You may also use PLOS’s free figure tool, NAAS, to help you prepare publication quality figures: https://journals.plos.org/plosone/s/figures#loc-tools-for-figure-preparation.

NAAS will assess whether your figures meet our technical requirements by comparing each figure against our figure specifications.

# Author comment: Author response to Decision Letter 1

**DOI:** 10.1371/journal.pone.0353122.r002

8 Jun 2026

Thank you for the your insightful feedback and the opportunity to revise our manuscript. Below we address point-by-point each of the reviewers suggestions for the manuscript. For easier understanding, we suggest looking at the version present in the "Response to Reviewers - Plos Mental ONE.docx" file attached to this submission, as it is color-coded for easier understanding of the answers and manuscript corrections.

Reviewer #1:

1. Sample characterization and recruitment

The inclusion and exclusion criteria should be described in greater detail. Given the inherent difficulty in accessing individuals with high levels of social withdrawal, the authors should further elaborate on their recruitment strategy and discuss whether it adequately captures individuals at the more severe end of the NEET/hikikomori spectrum. There is a potential risk of selection bias, particularly if participants were recruited through online platforms that may exclude the most socially withdrawn individuals.

Thank you for your comment. The inclusion and exclusion criteria were already detailed in the Participants section, but were rewritten for clarity as follows:

All participants were 18 or older, and each received 200 JPY for completing the survey. The survey was open to people with Japanese nationality and residence who were 18 or older and had previous experience with video games.

Exclusion criteria included participation in both survey rounds, failing at least one of two attention checks, leaving before fully completing the questionnaire, and not being born and raised in Japan.

Of our original 676 participants, 89 had invalid responses according to our exclusion criteria, and were thus excluded from the analysis.

Convenience sampling was conducted via Crowdworks because hikikomori and those at high risk of social isolation are traditionally avoidant of social-facing employment and are likely to seek remote, anonymous freelance work. This makes online sampling ideal for our target audience, as it potentially targets a larger population of hikikomori across the hikikomori risk spectrum. (For more information on the advantages of convenience sampling, please refer to page 19 of Landers, R. N., & Behrend, T. S. (2015). An inconvenient truth: Arbitrary distinctions between organizational, Mechanical Turk, and other convenience samples. Industrial and Organizational Psychology, 8(2), 142-164.

While recruitment was conducted via convenience sampling, the study maintained high internal validity by utilizing the validated NHR Scale as a psychometric filter to identify high-risk individuals, ensuring that the sample was representative of hikikomori profiles in Japan. This was added to the “Participants” sub-section of our Methods:

To address the potential bias of convenience sampling and ensure the quality of our data, we implemented the validated NEET-Hikikomori Risk (NHR) Scale to measure the hikikomori risk of participants. We found that results ranged between 44 and 153 points, from mild to more extreme risk of social isolation.

While recruitment was conducted via convenience sampling, the use of the validated NHR Scale allowed us to measure the risk levels within this specific digital demographic. Our sample mean (M=103.4) sits just below the proposed 104-point cutoff for ‘High Risk' (Uchida & Norasakkunkit, 2015), resulting in a distribution where 52.3% of participants are classified as high-risk. This distribution is consistent with our recruitment strategy, which specifically targeted individuals with gaming experience on a crowdsourcing platform —- two factors known to correlate with the NEET-hikikomori lifestyle (Stavropoulos et al., 2019). Consequently, our sample aligns with contemporary research showing that the hikikomori phenomenon increasingly affects a diverse, middle-aged digital workforce across genders (Yong et al., 2020).

Because our research goals focus on understanding the relationship between specific gaming preferences and the risk of social isolation, rather than estimating nationwide prevalence, the use of the validated NHR scale provides us the necessary tools for our comparative analysis without the requirement for population-wide weighting.

2. Clinical variables and potential confounders

Hikikomori is frequently associated with psychiatric conditions such as depression, social anxiety, and autism spectrum traits. The absence of these variables in the data collection is a notable limitation, as they may act as important confounders or mediators. The authors should justify this omission and explicitly acknowledge it in the discussion.

We appreciate this important point. We did not collect measures of associated psychiatric conditions. Besides the already mentioned autism spectrum traits in our current Limitations section, we have appended the following section to explicitly state the lack of measurements for the other mentioned conditions:

Because our focus was limited to this broader isolation risk, we did not examine comorbid conditions like autism, which can share psychiatric traits with severe withdrawal (Dell’Osso et al., 2023). Similarly, we did not examine participants' potential depression and social anxiety levels, which have been reported in high levels in previous hikikomori studies (Koyama et al., 2010). Thus, we did not investigate how these comorbidities might further influence gaming preferences. Future research should incorporate clinical samples and investigate a wider range of psychiatric conditions to better understand how formal diagnoses, such as hikikomori, specifically relate to game traits and player motivations.

3. Methodological transparency

Additional methodological details would improve reproducibility. Specifically:

Which statistical software and packages were used for data analysis?

How were missing data handled, particularly for incomplete questionnaire responses?

What was the average time required to complete the survey?

Thank you for your comment. The details on the software were included at the beginning of the Methods section:

All materials, data, and analyses were done with R for data formatting and analysis (version 2023.03.1+446); we utilized the ‘readxl' package for library import, ‘tidyverse' for plotting, ‘moments' to calculate skewness and kurtosis, ‘lm.beta' to calculate standardized β coefficients, ‘effectsize' to interpret the R² results, and ‘xtable' and ‘knitr' for table building. The source code and analysis …

Missing data and average completion time were mentioned in the Participants sub-section:

Exclusion criteria included having answered the questionnaire more than once (in the same round or across both survey rounds), failing at least one of two attention checks, leaving before fully completing the questionnaire, and not being born and raised in Japan.

[…]

The average completion time of the questionnaire was around 32 minutes.

4. Reporting of results

The abstract would benefit from a clearer presentation of the statistical significance and direction of the main findings. This would improve accessibility and ensure that key results are immediately interpretable.

Thank you for your comment. We have complemented the abstract to clearly state the connections between the relevant constructs and their interpretations:

After accounting for age, gender and extraversion, results showed that higher NHR score was associated with lower HEXAD Socialiser, lower HEXAD Philanthropist and lower Game Traits Social orientation scores, suggesting a negative association between NEET-Hikikomori risk and player traits related to socialisation. Furthermore, exploratory analysis showed that a higher NHR score was associated with lower scores in HEXAD Achiever and Game Traits Challenges orientation, suggesting NEET-Hikikomori risk has a negative correlation with challenges and achievement-related gameplay.

Furthermore, we have clarified this in the “Hypothesised analyses” sub-section to facilitate the understanding of the direction of our findings throughout the manuscript:

In other words, higher NHR score was associated with lower HEXAD Socialiser, lower HEXAD Philanthropist, and lower GTS Social Orientation scores.

A similar summary sentence was also included in the “Exploratory analysis” sub-section:

In other words, a higher NHR score was associated with lower scores in the HEXAD Free Spirit, HEXAD Achiever, HEXAD Player, Game Traits Narrative, and Game Traits Challenge constructs, even after accounting for age and gender.

5. Interpretation of findings

The conclusion that individuals at higher withdrawal risk are less socially engaged even in gaming environments may be somewhat unidimensional. Alternative interpretations should be considered. For example, a preference for solitary or low-pressure forms of engagement may represent a coping strategy rather than a deficit. Expanding the discussion to include this perspective would add important nuance.

Thank you for your comment. As the reviewer points out, there may be more reasons that could lead those at higher risk of social isolation to want to avoid social interactions in gaming environments. This has been reflected in the Discussion section:

It is important to consider, however, that this observed preference for lower social engagement should not be interpreted solely as a psychological deficit. Instead, such tendencies may represent a proactive coping strategy used by high-risk players to manage their emotional well-being in digital environments, particularly those that can lead to stressful situations. Previous research (Frommel, Johnson & Mandryk, 2023) found that “players who perceived their gaming communities as toxic also reported higher loneliness and lower need satisfaction of relatedness”. Considering these results, gravitating toward more solitary forms of engagement could be the way some players avoid the `vicious cycle' of toxicity found in some online gaming communities. One possible medium that could offer 'low-risk' experiences are narrative-driven Visual Novels, which remove the immediate pressure of direct social confrontation and the stress of negative feedback, while still offering the possibility of forming parasocial relationships with the characters in the stories. This allows players to feel like they are maintaining their ‘digital social capital' (Ellison, Steinfield, & Lampe, 2007) while protecting themselves from further social impairment.

6. Reliance on self-report measures

All variables were assessed via self-report, which introduces the possibility of reporting bias, particularly in sensitive domains such as social functioning. The authors should acknowledge this limitation more explicitly and consider the value of incorporating behavioral data (e.g., in-game activity logs, multiplayer participation, communication patterns) in future research.

Thank you for this insightful point. We understand that the exclusive use of self-report measures could be a bias-inducing limitation. However, given the reclusive nature of the high-risk NEET-hikikomori population, anonymous online surveys are currently one of the most effective methods to reach these individuals, as they often seek remote and anonymous environments. To mitigate this issue, we decided to utilize the NHR scale, which has been externally validated and shown to strongly correlate with actual behavioral indicators of social isolation. Nevertheless, we have updated our Limitations section to explicitly address this bias and propose the integration of behavioral data in future research.

A further limitation of this study is its exclusive reliance on self-report measures, which may be subject to reporting bias or social desirability, particularly in sensitive domains related to social withdrawal and impairment. The psychometric tools used, such as the NHR (Uchida & Norasakkunkit, 2015) and HEXAD (Diamond et al., 2015; Tondello et al., 2016) scales are validated for this population, but they do not capture real-time player behavior. Future research should investigate these psychometric findings using objective behavioral data, such as in-game activity logs, the frequency of multiplayer participation, and digital communication patterns between players. Incorporating such metrics could provide a more robust understanding of how the psychological dispositions identified in this study translate into actual engagement within virtual environments.

7. Granularity of gaming variables

The study would benefit from greater specificity regarding gaming behaviors and preferences (e.g., game genres, multiplayer versus single-player engagement, types of social interaction). This limitation should be acknowledged, as it constrains the practical implications of the findings.

Thank you for your comment. We agree that providing greater specificity regarding gaming behaviors, such as the balance of multiplayer versus single-player engagement and more specific game genres, would enrich the findings of this study. However, the primary aim of this research was to move beyond broad game genres, to instead identify the underlying psychological traits and motivational orientations of high-risk players. By focusing on stable traits (through measurements via the HEXAD and GT Scales), we can provide a more flexible theoretical foundation, which could be applied across various genres. For instance, our main finding that high-risk gamers have lower “Social” and “Challenge” orientations allows us to propose genre-independent design requirements, such as shifting focus from competition to cooperation, or implementing simple progression mechanics to avoid sudden shifts in the challenge level. We have updated the Limitations section to address this lack of granularity and discuss its implications for the study’s practical limitations:

The practical value of this study’s trait-based approach lies in its ability to derive genre-independent design requirements that are robust across various virtual environments. By identifying stable psychological dispositions —-such as the observed aversion to competition and high-difficulty tasks—- we can propose specific mechanics, like cooperative-only social loops and low-friction, RPG-like progression, that can be implemented regardless of whether the game `vessel' is an Action game or a Visual Novel, for example. These findings provide a strong, theory-driven foundation for needs-based design grounded in Self-Determination Theory (Ryan & Deci, 2000).

However, a limitation of the current study is the lack of granularity regarding specific, day-to-day gaming behaviors, such as a detailed breakdown of time spent in multiplayer versus single-player modes or the frequency of specific types of social interaction. While our trait-focused categories provide a stable roadmap, the absence of this behavioral data may impede immediate title-specific translational impact. Future research should therefore aim to capture these play patterns in more detail, refining the general psychological profiles into precise, highly specific design recommendations.

Furthermore, the practical value of this trait-based approach is shown by our ability to derive genre-independent design requirements, which can be seen in the following question.

8. Implications for intervention

While the manuscript highlights the relevance of tailoring game design to different player profiles, the implications for intervention remain somewhat underdeveloped. More concrete, theory-driven suggestions would strengthen the translational impact of the study.

Thank you for your suggestion. To strengthen the translational impact of our study, we have expanded the Discussion section to include the “Design suggestions and implications for interventions” sub-section. In it, we suggest concrete, theory-driven design implications grounded in the Self-Determination Theory (SDT) (Ryan & Deci, 2000). By mapping our trait-based findings to the SDT’s core needs for Competence, Autonomy and Relatedness, we propose that interventions for high-risk players could: (1) prioritize cooperative social mechanics over competition, to bypass social anxiety and fears of toxicity (which could help with players’ Relatedness needs); (2) utiliz

Attachment Submitted filename: Response to Reviewers - Plos Mental ONE.docx

# Peer review: Decision Letter 1

**DOI:** 10.1371/journal.pone.0353122.r003

**Authors:** Marc Potenza

18 Jun 2026

Social marginalization risk and its negative association with socialising preferences in Japanese gamers

PONE-D-26-12060R1

Dear Dr. Confessor,

We’re pleased to inform you that your manuscript has been judged scientifically suitable for publication and will be formally accepted for publication once it meets all outstanding technical requirements.

Within one week, you’ll receive an e-mail detailing the required amendments. When these have been addressed, you’ll receive a formal acceptance letter and your manuscript will be scheduled for publication.

An invoice will be generated when your article is formally accepted. Please note, if your institution has a publishing partnership with PLOS and your article meets the relevant criteria, all or part of your publication costs will be covered. Please make sure your user information is up-to-date by logging into Editorial Manager at Editorial Manager® and clicking the ‘Update My Information' link at the top of the page. For questions related to billing, please contact billing support.

If your institution or institutions have a press office, please notify them about your upcoming paper to help maximize its impact. If they’ll be preparing press materials, please inform our press team as soon as possible -- no later than 48 hours after receiving the formal acceptance. Your manuscript will remain under strict press embargo until 2 pm Eastern Time on the date of publication. For more information, please contact onepress@plos.org.

Kind regards,

Marc N Potenza

Academic Editor

PLOS One

Additional Editor Comments (optional):

Reviewers' comments:

Reviewer's Responses to Questions

**Comments to the Author**

1. If the authors have adequately addressed your comments raised in a previous round of review and you feel that this manuscript is now acceptable for publication, you may indicate that here to bypass the “Comments to the Author” section, enter your conflict of interest statement in the “Confidential to Editor” section, and submit your "Accept" recommendation.

Reviewer #1: All comments have been addressed

Reviewer #2: All comments have been addressed

**********

2. Is the manuscript technically sound, and do the data support the conclusions?

The manuscript must describe a technically sound piece of scientific research with data that supports the conclusions. Experiments must have been conducted rigorously, with appropriate controls, replication, and sample sizes. The conclusions must be drawn appropriately based on the data presented.

Reviewer #1: Yes

Reviewer #2: Yes

**********

3. Has the statistical analysis been performed appropriately and rigorously?

Reviewer #1: I Don't Know

Reviewer #2: Yes

**********

4. Have the authors made all data underlying the findings in their manuscript fully available?

The PLOS Data policy requires authors to make all data underlying the findings described in their manuscript fully available without restriction, with rare exception (please refer to the Data Availability Statement in the manuscript PDF file). The data should be provided as part of the manuscript or its supporting information, or deposited to a public repository. For example, in addition to summary statistics, the data points behind means, medians and variance measures should be available. If there are restrictions on publicly sharing data—e.g. participant privacy or use of data from a third party—those must be specified.

Reviewer #1: Yes

Reviewer #2: Yes

**********

5. Is the manuscript presented in an intelligible fashion and written in standard English?

PLOS ONE does not copyedit accepted manuscripts, so the language in submitted articles must be clear, correct, and unambiguous. Any typographical or grammatical errors should be corrected at revision, so please note any specific errors here.

Reviewer #1: Yes

Reviewer #2: Yes

**********

6. Review Comments to the Author

Please use the space provided to explain your answers to the questions above. You may also include additional comments for the author, including concerns about dual publication, research ethics, or publication ethics. (Please upload your review as an attachment if it exceeds 20,000 characters)

Reviewer #1: The authors addressed all my suggestions in the revised version of the manuscript, as described in the point-by-point response to reviewers.

Reviewer #2: The authors have made substantial revisions and significantly improved the overall quality of this manuscript. All of my concerns have been adequately addressed. I have no further comments.

**********

7. PLOS authors have the option to publish the peer review history of their article (what does this mean?). If published, this will include your full peer review and any attached files.

If you choose “no”, your identity will remain anonymous but your review may still be made public.

**Do you want your identity to be public for this peer review?**  For information about this choice, including consent withdrawal, please see our Privacy Policy.

Reviewer #1: No

Reviewer #2: **Yes:** Kunru Song

**********

# Editor report: Acceptance letter

**DOI:** 10.1371/journal.pone.0353122.r004

**Authors:** Marc Potenza

PONE-D-26-12060R1

PLOS One

Dear Dr. Confessor,

I'm pleased to inform you that your manuscript has been deemed suitable for publication in PLOS One. Congratulations! Your manuscript is now being handed over to our production team.

At this stage, our production department will prepare your paper for publication. This includes ensuring the following:

* All references, tables, and figures are properly cited

* All relevant supporting information is included in the manuscript submission,

* There are no issues that prevent the paper from being properly typeset

You will receive further instructions from the production team, including instructions on how to review your proof when it is ready. Please keep in mind that we are working through a large volume of accepted articles, so please give us a few days to review your paper and let you know the next and final steps.

Lastly, if your institution or institutions have a press office, please let them know about your upcoming paper now to help maximize its impact. If they'll be preparing press materials, please inform our press team within the next 48 hours. Your manuscript will remain under strict press embargo until 2 pm Eastern Time on the date of publication. For more information, please contact onepress@plos.org.

You will receive an invoice from PLOS for your publication fee after your manuscript has reached the completed accept phase. If you receive an email requesting payment before acceptance or for any other service, this may be a phishing scheme. Learn how to identify phishing emails and protect your accounts at https://explore.plos.org/phishing.

If we can help with anything else, please email us at customercare@plos.org.

Thank you for submitting your work to PLOS ONE and supporting open access.

Kind regards,

PLOS ONE Editorial Office Staff

on behalf of

Dr. Marc N Potenza

Academic Editor

PLOS One
